# Pulsed electrolysis: enhancing primary benzylic C(sp^3^)–H nucleophilic fluorination†

**DOI:** 10.1039/d3qo01865b

**Published:** 2023-12-13

**Authors:** Alexander P. Atkins, Atul K. Chaturvedi, Joseph A. Tate, Alastair J. J. Lennox

**Affiliations:** a School of Chemistry, University of Bristol Cantock's Close BS8 1TS Bristol UK a.lennox@bristol.ac.uk; b Jealott's Hill International Research Centre, Syngenta Jealott's Hill Bracknell RG426EY UK

## Abstract

Electrosynthesis is an efficient and powerful tool for the generation of elusive reactive intermediates. The application of alternative electrolysis waveforms provides a new level of control for dynamic redox environments. Herein, we demonstrate that pulsed electrolysis provides a favourable environment for the generation and fluorination of highly unstable primary benzylic cations from C(sp^3^)–H bonds. By introduction of a *t*_off_ period, we propose this waveform modulates the electrical double layer to improve mass transport and limit over-oxidation.

## Abstract 2

Pulsed electrolysis waveforms benefit primary benzylic cation generation and fluorination.

## Graphical abstract

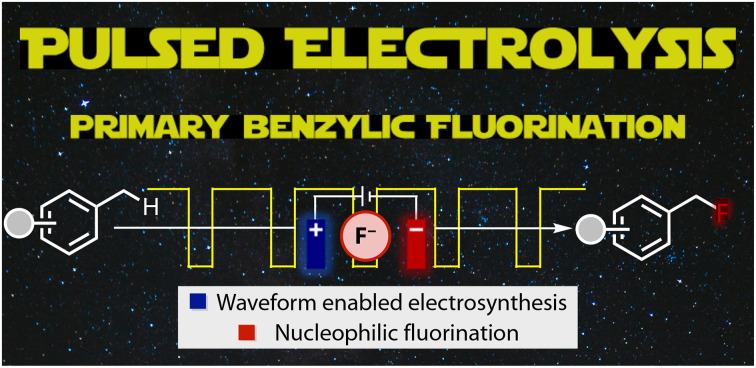

Organic electrochemistry offers unique control of redox reactions through the ability to accurately control potentials and rates, which has several advantages for selectivity and sustainability in synthesis.^1–4^ Electrochemical anodic oxidation and cathodic reduction is demonstrated as a means of generating highly reactive intermediates,^5–8^ including, ionic species, radicals (cations/anions), and reactive metal complexes (Fig. 1A). These intermediates are useful and important for myriad synthetic transformations,^5,9^ and can be uniquely generated from starting materials without pre-functionalisation using electrochemistry.

**Fig. 1:**
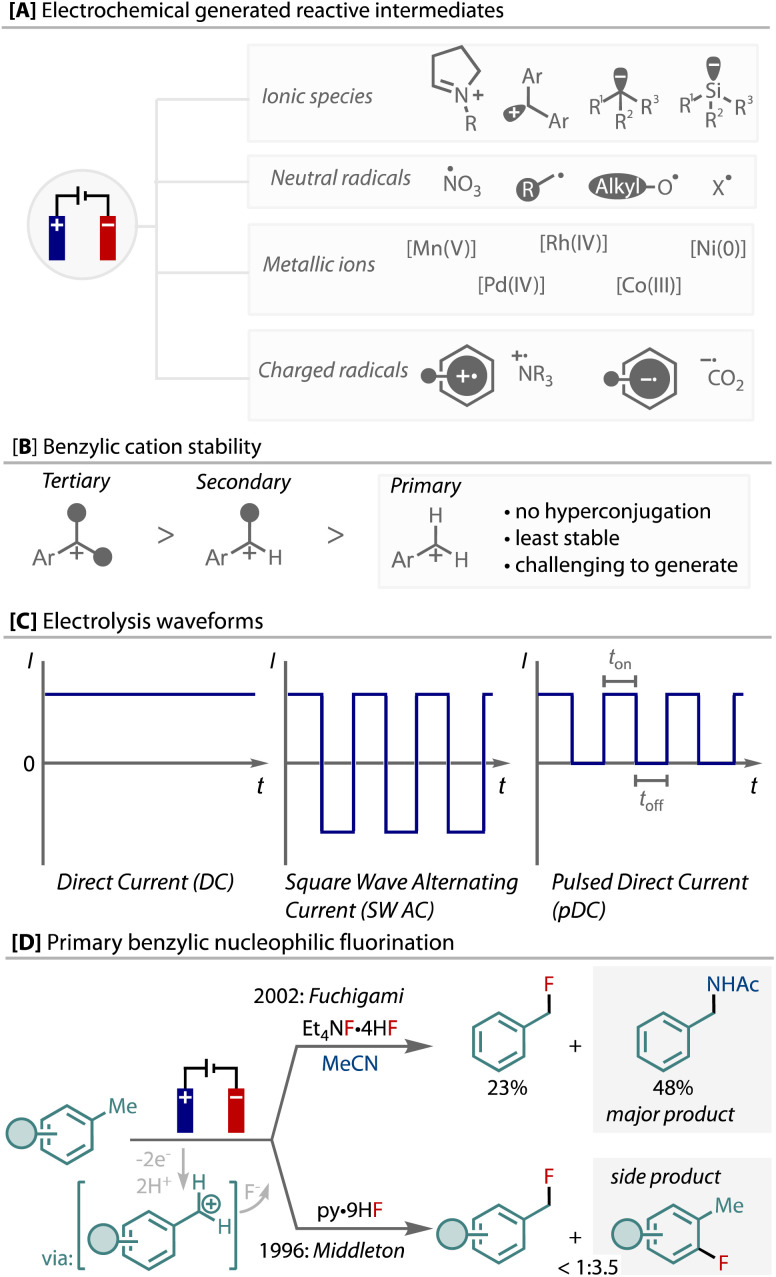
A: Electrochemical generation of highly reactive intermediates; B: benzylic cations as elusive intermediates; C: alternative waveforms in electrochemistry; D: previous examples of electochemical primary benzylic C–H fluorination.

An elusive and particularly reactive intermediate is the primary benzylic cation. Electrochemical oxidation has been demonstrated as an efficient means of producing benzylic cations from benzylic C(sp^3^)–H bonds, *via* a sequential electron-transfer, proton-transfer, electron-transfer (ET/PT/ET) sequence.^10–17^ Without the stabilisation that hyperconjugation provides, primary are less stable than secondary, which are less stable than tertiary benzylic cations, Fig. 1B. Hence, in contrast to secondary and tertiary, the generation of primary benzylic cations by electrochemical oxidation and their functionalisation has been reported very few times, and as such is limited to the use of highly electron-rich substrates, solvent level nucleophile, or a cation-pool strategy.^18–23^

The introduction of alternative electrolysis waveforms has been demonstrated as a tool for facilitating unique reactivity (Fig. 1C).^24–26^ For example, alternating polarity has enabled new reactivity and selectivity, including reactions of short-lived species *via* paired-electrolysis,^27^ unique chemoselectivity for reductions,^28,29^ or selective control over one- or two-electron reactivity,^30^ demonstrating the additional level of control that can be enabled by dynamic redox environments using an electrochemical strategy.

Within this vein, we were interested to investigate how alternative waveforms might influence the generation and functionalisation of primary benzylic cations. Despite the strategically facile nature of generating reactive species electrochemically, it does not come without challenges. These include undesired side-reactions and electrode-grafting/fouling processes that add to the difficulty and complexity.^31^ Specifically, we hypothesized that dynamic manipulation of the electrical double layer may aid their desired functionalization and avoid side-reactions. This unique electrode-surface environment can be altered with the applied potential (fixed or moving), electrode material, electrolyte composition and mass transfer regimes, which all influence the outcome of the reaction.^32–38^

We elected to conduct these fundamental studies on the formation and functionalisation of primary benzylic cations from C(sp^3^)–H bonds using fluorination as a test reaction. Benzylic and aliphatic fluorides have been realised *via* photochemical,^39–43^ radical^44–50^ and metal-catalysed strategies^51–55^ from C–H bonds, as well as decarboxylative routes.^56–60^ However, the nucleophilic fluorination of benzylic C(sp^3^)–H bonds *via* cationic intermediates is a highly challenging transformation; current methods are limited and almost entirely focussed on secondary and tertiary benzylic C(sp^3^)–H bonds,^42,43,61,62^ with only two prior reports showing examples of primary, Fig. 1D.^20,21^ Fuchigami demonstrated only toluene as a suitable substrate, in which Ritter-amination competed, and Middleton demonstrated that benzylic fluorination competes with C(sp^2^)–H ring fluorination on a set of electron-poor toluene derivatives. In addition, unlike secondary and tertiary benzylic fluorides,^52,61,63^ most primary benzyl fluorides are stable and can be isolated. Herein, we describe our studies on this reaction, in which we have discovered that the unusual action of either pulsing direct current (pDC) or pulsed step constant potential (pSCP) is able to achieve enhanced yield and selectivity in this highly challenging transformation.

Our exploration studies employed biphenyl 1a due to its low volatility and ease of monitoring by ^19^F NMR, Fig. 2. Variation of the fluoride source, equivalents, solvent, temperature and concentration afforded the corresponding benzyl fluoride 2a in 34% yield after passing 2*F* and stirring at 1000 rpm, Fig. 2A. The outcome remained sensitive to the fluoride, with Et_3_N·3HF identified as the best source, serving as the supporting electrolyte, fluoride source and a source of proton as the oxidant for the cathodic counter-electrode process.^64^ A divided cell did not improve the yield.

**Fig. 2:**
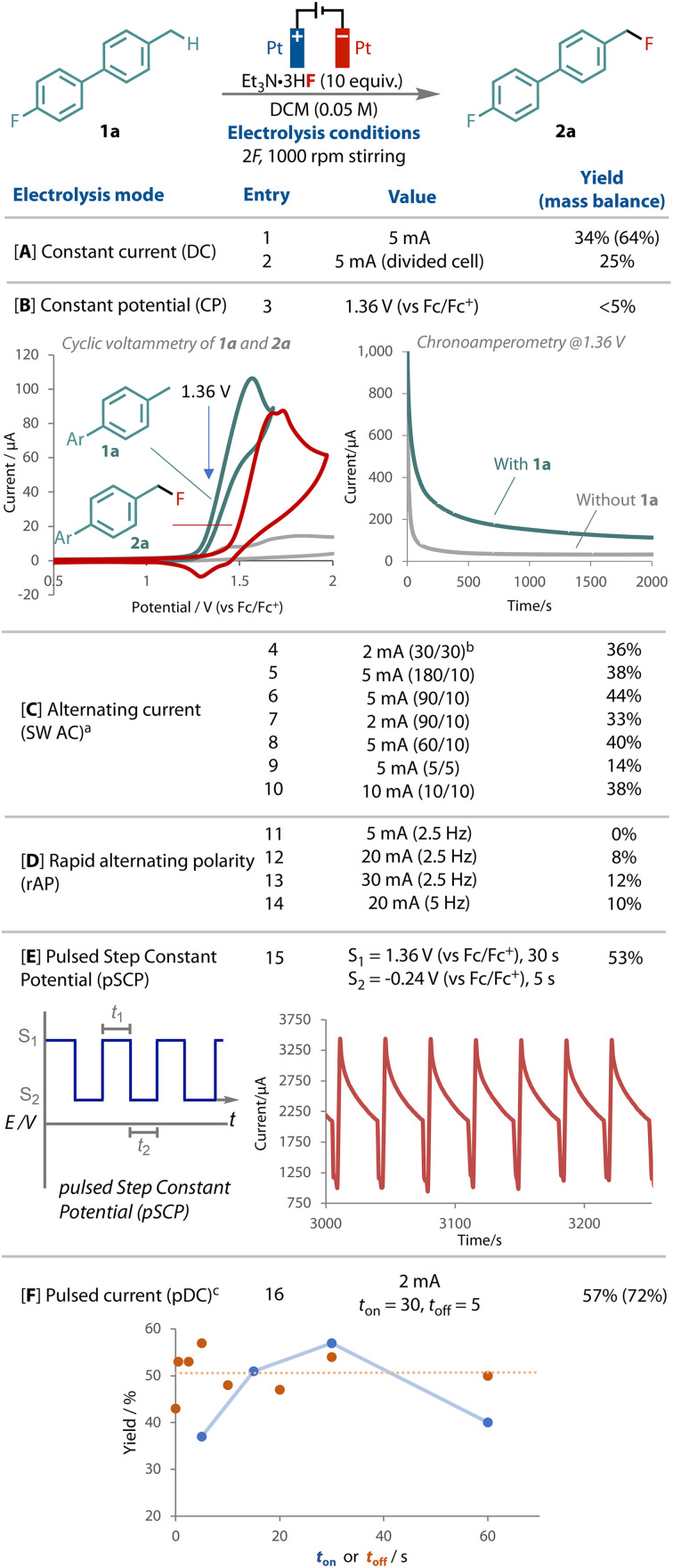
Summary of electrochemical waveforms tested on the transformation of 1a to 2a. NMR yields. ^*a*^ SW = square wave; ^*b*^ the square wave period sequence, seconds. *^c^* 2,6-lutidinium.HBF4 (0.1 M) added.

Electrode fouling and grafting were indeed observed on the anode and cathode during these preliminary studies,^4^ resulting in a reduced mass balance of 64% with DC. Unsuccessful attempts to characterise discrete surface species support substrate decomposition through over-oxidation. As the difference in the onset potential between substrate 1a and product 2a is small, Fig. 2B, we elected to apply a constant potential of 1.36 V (*vs*. Fc/Fc^+^), which is specific for 1a oxidation but below the onset of product 2a. Surprisingly however, although the current response was initially at levels expected for the concentration and electrode surface area, it rapidly dropped off to the μA range, even with rapid stirring, and resulted in only trace 2a, Fig. 2B. The observed current was only marginally greater than the non-faradaic background current observed when no 1a was present. This effect may be due to the formation of an insulating film,^21,65^ possibly from electrode polarisation, which limits access of substrate 2a to the electrode surface.

The use of a square wave alternating current (AC) was tested by switching the polarity between the two electrodes and holding for a certain length of time, Fig. 2C. Several different period-lengths were tested,^64^ and a 10% yield improvement was observed compared to direct current. We also tested rapid alternating current (rAP), Fig. 2D, following several recent successful examples that have led to unique selectivity.^28–30^ However, unfortunately, with all the currents and frequencies that we applied, lower yields were observed in all cases.

Interestingly, significant improvement was observed when a pulsed step constant potential (pSCP) electrolysis was performed, in which two levels of constant potential were cycled (*S*_1_ = 1.36 V *t*_1_ = 30 s, *S*_2_ = −0.24 V *t*_2_ = 5 s), Fig. 2E. This method afforded the greatest improvement in yield, entry 15. This result was especially curious considering that when 1.36 V was run continuously only trace product was observed (entry 3). This waveform has been shown to improve reactant mass transport and concentrations at the working electrode in reductive acrylonitrile dimerization,^66^ and has also been reported previously in CO_2_ reductive electrolysis.^67^ The introduction of a second step potential serves as a resting potential, which limits faradaic processes.

Analysis of the chronoamperometry output during the step potential experiment showed high currents at the start of each *S*_1_ period. The current decreased during this period, before rapidly decreasing during the resting, *S*_2_, period when lower potential is applied, Fig. 2E. The next *S*_1_ period restores the higher level of observed current, and the cycle repeats. What is most significant to note here is that compared to the constant potential (chronoamperometry) experiment, inclusion of the resting, *S*_2_, period induces substantially higher currents, both at the peak and also throughout the *S*_1_ period (Fig. 2E *vs.* B). The sharp reduction in observed current during *S*_2_ suggests periods of suppressed faradaic reactivity are occurring and facilitating the improvement in reactivity. This further highlights the fragility of the relationship between electrical double layer and reaction outcome.

A mean average of 2 mA was observed during the step electrolysis reaction, therefore for practicality purposes we elected to use a pulsed direct current electrolysis (pDC) (*t*_on_*I* = 2 mA; *t*_off_*I* = 0 mA) as a means of cycling through productive and resting cycles. Several different *t*_on_ and *t*_off_ period lengths were tested, Fig. 2F, where it was found that pulsing at 2 mA for 30 seconds (*t*_on_) followed by a 5 seconds period where no current was applied (*t*_off_) yielded reliably enhanced yields of the primary benzylic fluorination product, entry 16.

Analysis of the different pulsing sequencies tested with pDC, Fig. 2F, showed that, while the inclusion of a *t*_off_ period clearly improved the reaction outcome, adjustment of its duration did not have a significant impact, as demonstrated by the flat line of best fit, Fig. 2F (orange line). With regards to the *t*_on_ period, 30 seconds proved to be optimal, with longer and shorter periods showing a decline in the yield of 2a (blue points).

We sought to validate the difference between pDC and DC by applying the two different waveforms across a selection of other primary benzylic substrates and monitoring the yield, Fig. 3. To provide the greatest confidence in the results, we performed each reaction in triplicate to verify the improvement that pulsed current electrolysis provides. These studies revealed that an enhanced yield was observed when applying PC compared to DC, in every substrate tested that gave synthetically useful yields. For example, for model substrate 1a an average enhancement of 15% was observed with PC, while other substrates (1b–g), including those with halide, carbonate and trifluoromethyl substituents gave equally significant improvements to the yield for these primary benzyl fluorides. Only in low yielding substrates (<20%) was there no significant difference between PC and DC observed. Pulsed current also led to similar benefits for the fluorination of secondary benzylic substrates too, as exhibited by 1h and 1i, highlighting that this effect also relates to the fluorination of secondary benzylic cations.

**Fig. 3:**
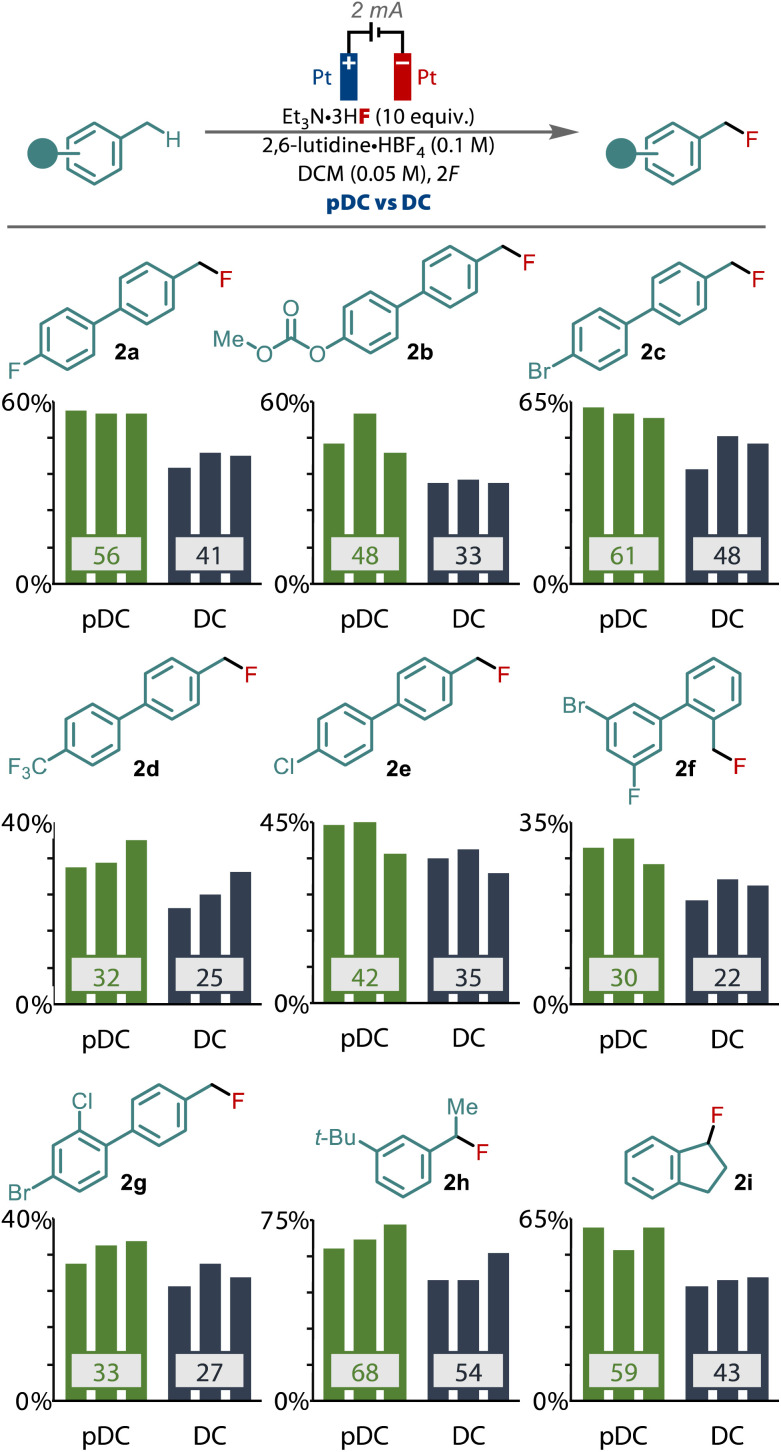
Comparison of pDC *vs*. DC electrolysis for a selection of primary and secondary benzylic substrates.

To confirm the differences between constant potential and pulsed step constant potential electrolysis, we elected to switch between both techniques in a single reaction of 1a to 2a, and take aliquots to gauge the corresponding level of product formation that each waveform is responsible for, Fig. 4A. Consistent with what was observed during their exclusive use (Fig. 2E *vs*. B), product formation was only observed during periods where step potential was applied and the reaction completely stalled when constant potential was applied. Interestingly, the consumption of 1a still occurred, albeit in an attenuated rate, during the periods of constant potential, demonstrating the sensitive nature of the intermediates formed. To the best of our knowledge, this remarkable effect has not been previously reported, and clearly demonstrates the importance of controlling the electrode/solution interface.

**Fig. 4:**
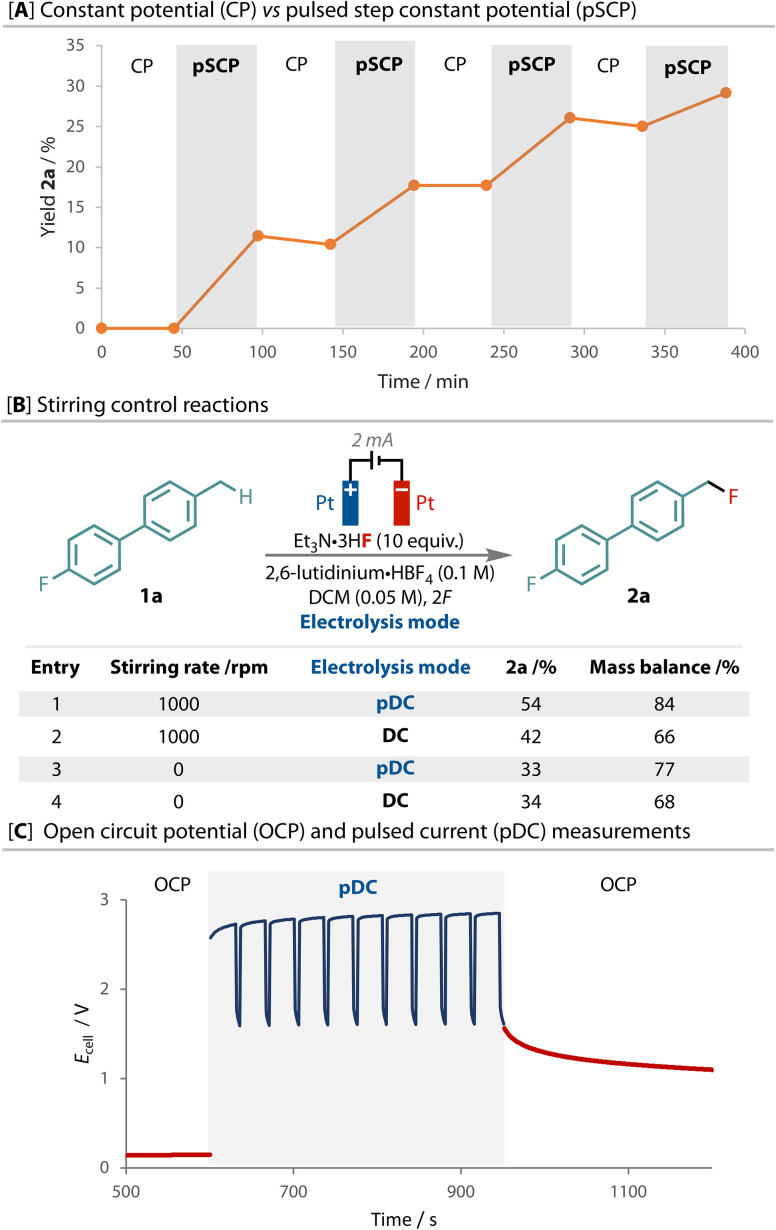
A: Sequential direct and pulsed potential experiment showing how the yield of 2a only increases during the pulsed periods, but the loss of substrate 1a occurs under both regimes, albeit faster in pulsed. B: The effect of stirring on the direct and pulsed potential regimes; C: measurement of the open circuit potential before and after PP electrolysis.

Despite rapid stirring in all experiments presented thus far, we proposed this effect to be due to limitations of mass transport within a highly ordered fluoride-containing electrical double-layer. Pulsed and direct current were therefore compared under different stirring regimes to add insight to this hypothesis. Both the yield and mass balance are higher with pulsed current than with direct current when the reaction is stirred rapidly, Fig. 4B. However, without stirring, although the mass balance is still different, the yield of product 2a was the same with both pulsed and direct currents. Hence, when the solution at the electrode is not replaced, pulsing has no effect compared to constant current. This evidence highlights the importance of stirring to the *t*_off_ period in dispersing charged species from the electrode surface and replenishing it with substrate for further reaction.

The dispersion of charged species during the *t*_off_ periods can be observed by measuring the cell potential during periods when no current is flowing through the cell *via* open-circuit potential (OCP) measurements before and after electrolysis, Fig. 4C. The OCP (*E*_cell_, red line) remained low and constant before electrolysis. During pulsed current (pDC) electrolysis (blue line) a high *E*_cell_ was maintained and then dropped during *t*_off_, but to a level much higher than the original OCP. The difference between the *t*_on_ and *t*_off_ periods (*E*^max–min^_cell_) was only *ca.* 1 V, compared to a *E*^max-OCP^_cell_ of almost 3 V. Despite the absence of applied current during these *t*_off_ periods, the observed *E*_cell_ does not return to pre-electrolysis levels indicating electrical activity through the delayed dispersion of charged species. This point is emphasised after electrolysis, as the OCP follows the final *t*_off_ period and only slowly trends toward the original OCP.

These combined results suggest that the pulse sequence, specifically the *t*_off_ period, is important to improve mass transport limitations through the fluorinated electrical double-layer that are observed during the constant potential experiment. In effect, pulsing increases mass transport and hence the concentration of substrate at the electrode surface,^68^ but also decreases the degree of over-oxidation and decomposition.

In conclusion, we have demonstrated how the use of pulsed current (pDC) electrolysis can provide enhancements in yield and selectivity for the challenging primary benzylic C(sp^3^)–H fluorination reaction. By including a *t*_off_ period a direct current or constant potential experiment, we observed consistently and reliably higher yields than the corresponding direct current electrolysis. Our analysis suggests that this approach allows for modulation of the electrical double layer to improve mass transport in this reaction, replenish substrate at the electrode surface, reduce over oxidation and decomposition and improve reaction efficiency. More broadly, this work demonstrates the increased control that is possible for the generation and functionalisation of reactive intermediates when using alternative electrolysis waveforms, and therefore should find further application in the field of synthetic organic electrosynthesis.

## Conflicts of interest

There are no conflicts to declare.

## Supplementary Material

QO-011-D3QO01865B-s001

## References

[cit1] Fuchigami T., Inagi S. (2020). Recent Advances in Electrochemical Systems for Selective Fluorination of Organic Compounds. Acc. Chem. Res..

[cit2] Frontana-Uribe B. A., Little R. D., Ibanez J. G., Palma A., Vasquez-Medrano R. (2010). Organic electrosynthesis: a promising green methodology in organic chemistry. Green Chem..

[cit3] Wiebe A., Gieshoff T., Möhle S., Rodrigo E., Zirbes M., Waldvogel S. R. (2018). Electrifying Organic Synthesis. Angew. Chem., Int. Ed..

[cit4] Heard D. M., Lennox A. J. J. (2020). Electrode Materials in Modern Organic Electrochemistry. Angew. Chem., Int. Ed..

[cit5] Yan M., Kawamata Y., Baran P. S. (2017). Synthetic Organic Electrochemical Methods Since 2000: On the Verge of a Renaissance. Chem. Rev..

[cit6] Malapit C. A., Prater M. B., Cabrera-Pardo J. R., Li M., Pham T. D., McFadden T. P., Blank S., Minteer S. D. (2022). Advances on the Merger of Electrochemistry and Transition Metal Catalysis for Organic Synthesis. Chem. Rev..

[cit7] El Gehani A. A. M. A., Maashi H. A., Harnedy J., Morrill L. C. (2023). Electrochemical generation and utilization of alkoxy radicals. Chem. Commun..

[cit8] Zhang W., Guan W., Martinez Alvarado J. I., Novaes L. F. T., Lin S. (2023). Deep Electroreductive Chemistry: Harnessing Carbon- and Silicon-Based Reactive Intermediates in Organic Synthesis. ACS Catal..

[cit9] Kärkäs M. D. (2018). Electrochemical strategies for C–H functionalization and C–N bond formation. Chem. Soc. Rev..

[cit10] Gao H., Chen X., Wang P.-L., Shi M.-M., Shang L.-L., Guo H.-Y., Li H., Li P. (2022). Electrochemical benzylic C–H arylation of xanthenes and thioxanthenes without a catalyst and oxidant. Org. Chem. Front..

[cit11] Tang S., Guillot R., Grimaud L., Vitale M. R., Vincent G. (2022). Electrochemical Benzylic C–H Functionalization with Isocyanides. Org. Lett..

[cit12] Zhang S., Li Y., Wang T., Li M., Wen L., Guo W. (2022). Electrochemical Benzylic C(sp 3)–H Isothiocyanation. Org. Lett..

[cit13] Atkins A. P., Rowett A. C., Heard D. M., Tate J. A., Lennox A. J. J. (2022). Electrochemical Benzylic C(sp 3)–H Acyloxylation. Org. Lett..

[cit14] Hou Z., Liu D., Xiong P., Lai X., Song J., Xu H. (2021). Site–Selective Electrochemical Benzylic C–H Amination. Angew. Chem., Int. Ed..

[cit15] Oliva M., Coppola G. A., Van der Eycken E. V., Sharma U. K. (2021). Photochemical and Electrochemical Strategies towards Benzylic C–H Functionalization: A Recent Update. Adv. Synth. Catal..

[cit16] Liu D., Zhang Z., Yu J., Chen H., Lin X., Li M., Wen L., Guo W. (2022). Site-selective electrochemical thiocyanation of benzylic C-H bonds. Org. Chem. Front..

[cit17] He G., Li Y., Zhou S., Yang X., Shang A., Wang Y., Liu H., Zhou Y. (2022). A Facile Electrochemical Strategy for the Azidation of Benzylic C(sp 3)–H Bonds. Eur. J. Org. Chem..

[cit18] Motsch B. J., Wengryniuk S. E. (2022). Site-Selective Synthesis of N-Benzyl 2,4,6-Collidinium Salts by Electrooxidative C–H Functionalization. Org. Lett..

[cit19] Wang X.-W., Deng Y., Li R.-X., Lv J.-F., Fu M.-Q.-H., Guan Z., Zhao Y.-N., He Y.-H. (2023). Electrochemical Direct Formyloxylation of Benzylic C(sp 3)–H with DMF. ACS Sustainable Chem. Eng..

[cit20] Tajima T., Ishii H., Fuchigami T. (2002). Anodic benzylic fluorination of toluene, ethylbenzene, and cumene derivatives. Electrochem. Commun..

[cit21] Lee S. M., Roseman J. M., Blair Zartman C., Morrison E. P., Harrison S. J., Stankiewicz C. A., Middleton W. J. (1996). Selective electrolytic fluorinations in 70% HF/30% pyridine. J. Fluor. Chem..

[cit22] Zhang L., Fu Y., Shen Y., Liu C., Sun M., Cheng R., Zhu W., Qian X., Ma Y., Ye J. (2022). Ritter-type amination of C(sp3)-H bonds enabled by electrochemistry with SO42−. Nat. Commun..

[cit23] Hayashi R., Shimizu A., Yoshida J.-I. (2016). The Stabilized Cation Pool Method: Metal- and Oxidant-Free Benzylic C–H/Aromatic C–H Cross-Coupling. J. Am. Chem. Soc..

[cit24] Rodrigo S., Gunasekera D., Mahajan J. P., Luo L. (2021). Alternating current electrolysis for organic synthesis. Curr. Opin. Electrochem..

[cit25] Jamshidi M., Fastie C., Hilt G. (2022). Applications of Alternating Current/Alternating Potential Electrolysis in Organic Synthesis. Synthesis.

[cit26] Zeng L., Wang J., Wang D., Yi H., Lei A. (2023). Comprehensive Comparisons between Directing and Alternating Current Electrolysis in Organic Synthesis. Angew. Chem., Int. Ed..

[cit27] Rodrigo S., Um C., Mixdorf J. C., Gunasekera D., Nguyen H. M., Luo L. (2020). Alternating current electrolysis for organic electrosynthesis: Trifluoromethylation of (hetero)arenes. Org. Lett..

[cit28] Kawamata Y., Hayashi K., Carlson E., Shaji S., Waldmann D., Simmons B. J., Edwards J. T., Zapf C. W., Saito M., Baran P. S. (2021). Chemoselective Electrosynthesis Using Rapid Alternating Polarity. J. Am. Chem. Soc..

[cit29] Hayashi K., Griffin J., Harper K. C., Kawamata Y., Baran P. S. (2022). Chemoselective (Hetero)Arene Electroreduction Enabled by Rapid Alternating Polarity. J. Am. Chem. Soc..

[cit30] Gunasekera D., Mahajan J. P., Wanzi Y., Rodrigo S., Liu W., Tan T., Luo L. (2022). Controlling One- or Two-Electron Oxidation for Selective Amine Functionalization by Alternating Current Frequency. J. Am. Chem. Soc..

[cit31] Lennox A. J. J., Nutting J. E., Stahl S. S. (2018). Selective electrochemical generation of benzylic radicals enabled by ferrocene-based electron-transfer mediators. Chem. Sci..

[cit32] Frey D. A., Hari Krishna Reddy S., Moeller K. D. (1999). Intramolecular Anodic Olefin Coupling Reactions: The Use of Allylsilane Coupling Partners with Allylic Alkoxy Groups. J. Org. Chem..

[cit33] Xiong P., Long H., Song J., Wang Y., Li J.-F., Xu H.-C. (2018). Electrochemically Enabled Carbohydroxylation of Alkenes with H 2 O and Organotrifluoroborates. J. Am. Chem. Soc..

[cit34] Kulisch J., Nieger M., Stecker F., Fischer A., Waldvogel S. R. (2011). Efficient and Stereodivergent Electrochemical Synthesis of Optically Pure Menthylamines. Angew. Chem., Int. Ed..

[cit35] Edinger C., Grimaudo V., Broekmann P., Waldvogel S. R. (2014). Stabilizing Lead Cathodes with Diammonium Salt Additives in the Deoxygenation of Aromatic Amides. ChemElectroChem.

[cit36] Subramanian K., Yedage S. L., Bhanage B. M. (2017). An Electrochemical Method for Carboxylic Ester Synthesis from N -Alkoxyamides. J. Org. Chem..

[cit37] Hioki Y., Costantini M., Griffin J., Harper K. C., Merini M. P., Nissl B., Kawamata Y., Baran P. S. (2023). Overcoming the limitations of Kolbe coupling with waveform-controlled electrosynthesis. Science.

[cit38] Moeller K. D. (2016). Anodic olefn coupling reactions: A mechanism driven approach to the development of new synthetic tools. Electrochem. Soc. Interface.

[cit39] Bloom S., McCann M., Lectka T. (2014). Photocatalyzed Benzylic Fluorination: Shedding “Light” on the Involvement of Electron Transfer. Org. Lett..

[cit40] Xia J.-B., Zhu C., Chen C. (2013). Visible Light-Promoted Metal-Free C–H Activation: Diarylketone-Catalyzed Selective Benzylic Mono- and Difluorination. J. Am. Chem. Soc..

[cit41] Yakubov S., Barham J. P. (2020). Photosensitized direct C–H fluorination and trifluoromethylation in organic synthesis. Beilstein J. Org. Chem..

[cit42] Leibler I. N., Tekle-Smith M. A., Doyle A. G. (2021). A general strategy for C(sp3)–H functionalization with nucleophiles using methyl radical as a hydrogen atom abstractor. Nat. Commun..

[cit43] Zhang Y., Fitzpatrick N. A., Das M., Bedre I. P., Yayla H. G., Lall M. S., Musacchio P. Z. (2022). A photoredox-catalyzed approach for formal hydride abstraction to enable C –H functionalization with nucleophilic partners (F, C, O, N, and Br/Cl). Chem. Catal..

[cit44] Hua A. M., Mai D. N., Martinez R., Baxter R. D. (2017). Radical C–H Fluorination Using Unprotected Amino Acids as Radical Precursors. Org. Lett..

[cit45] Takahira Y., Chen M., Kawamata Y., Mykhailiuk P., Nakamura H., Peters B. K., Reisberg S. H., Li C., Chen L., Hoshikawa T., Shibuguchi T., Baran P. S. (2019). Electrochemical C(sp3)–H Fluorination. Synlett.

[cit46] Madani A., Anghileri L., Heydenreich M., Möller H. M., Pieber B. (2022). Benzylic Fluorination Induced by a Charge-Transfer Complex with a Solvent-Dependent Selectivity Switch. Org. Lett..

[cit47] Yakubov S., Stockerl W. J., Tian X., Shahin A., Mandigma M. J. P., Gschwind R. M., Barham J. P. (2022). Benzoates as photosensitization catalysts and auxiliaries in efficient, practical, light-powered direct C(sp 3)–H fluorinations. Chem. Sci..

[cit48] Xu P., Guo S., Wang L., Tang P. (2014). Silver–Catalyzed Oxidative Activation of Benzylic C-H Bonds for the Synthesis of Difluoromethylated Arenes. Angew. Chem., Int. Ed..

[cit49] Danahy K. E., Cooper J. C., Van Humbeck J. F. (2018). Benzylic Fluorination of Aza–Heterocycles Induced by Single–Electron Transfer to Selectfluor. Angew. Chem., Int. Ed..

[cit50] Chatalova-Sazepin C., Hemelaere R., Paquin J.-F., Sammis G. (2015). Recent Advances in Radical Fluorination. Synthesis.

[cit51] Bloom S., Pitts C. R., Woltornist R., Griswold A., Holl M. G., Lectka T. (2013). Iron(ii)-Catalyzed Benzylic Fluorination. Org. Lett..

[cit52] Vasilopoulos A., Golden D. L., Buss J. A., Stahl S. S. (2020). Copper-Catalyzed C–H Fluorination/Functionalization Sequence Enabling Benzylic C–H Cross Coupling with Diverse Nucleophiles. Org. Lett..

[cit53] Hintz H., Bower J., Tang J., LaLama M., Sevov C., Zhang S. (2023). Copper-catalyzed electrochemical C–H fluorination. Chem. Catal..

[cit54] Liu W., Groves J. T. (2013). Manganese–Catalyzed Oxidative Benzylic C–H Fluorination by Fluoride Ions. Angew. Chem., Int. Ed..

[cit55] McMurtrey K. B., Racowski J. M., Sanford M. S. (2012). Pd-Catalyzed C–H Fluorination with Nucleophilic Fluoride. Org. Lett..

[cit56] Leech M. C., Nagornîi D., Walsh J. M., Kiaku C., Poole D. L., Mason J., Goodall I. C. A., Devo P., Lam K. (2023). eFluorination Using Cheap and Readily Available Tetrafluoroborate Salts. Org. Lett..

[cit57] Kiaku C., Martinage D., Sicim Y., Leech M. C., Walsh J. M., Poole D. L., Mason J., Goodall I. C. A., Devo P., Lam K. (2023). eFluorination of Activated Alcohols Using Collidinium Tetrafluoroborate. Org. Lett..

[cit58] Webb E. W., Park J. B., Cole E. L., Donnelly D. J., Bonacorsi S. J., Ewing W. R., Doyle A. G. (2020). Nucleophilic (Radio)Fluorination of Redox-Active Esters via Radical-Polar Crossover Enabled by Photoredox Catalysis. J. Am. Chem. Soc..

[cit59] Ventre S., Petronijevic F. R., MacMillan D. W. C. (2015). Decarboxylative Fluorination of Aliphatic Carboxylic Acids via Photoredox Catalysis. J. Am. Chem. Soc..

[cit60] Mizuta S., Stenhagen I. S. R., O'Duill M., Wolstenhulme J., Kirjavainen A. K., Forsback S. J., Tredwell M., Sandford G., Moore P. R., Huiban M., Luthra S. K., Passchier J., Solin O., Gouverneur V. (2013). Catalytic Decarboxylative Fluorination for the Synthesis of Tri- and Difluoromethyl Arenes. Org. Lett..

[cit61] Stangier M., Scheremetjew A., Ackermann L. (2022). Chemo– and Site–Selective Electro–Oxidative Alkane Fluorination by C(sp 3)–H Cleavage. Chem. – Eur. J..

[cit62] Yamashita K., Fujiwara Y., Hamashima Y. (2023). Amide-Ligand-Promoted Silver-Catalyzed C–H Fluorination via Radical/Polar Crossover. J. Org. Chem..

[cit63] Liu W., Huang X., Groves J. T. (2013). Oxidative aliphatic C-H fluorination with manganese catalysts and fluoride ion. Nat. Protoc..

[cit64] See ESI† for details

[cit65] Savett S. C., Lee S. M., Bradley A. Z., Kneizys S. P., Lobue J. M., Middleton W. J. (1993). Microscale Electrolytic Fluorinations of 4-Nitrotoluene - Cell Construction, Computer Monitor and Control, and Chemistry. Microchem. J..

[cit66] Blanco D. E., Lee B., Modestino M. A. (2019). Optimizing organic electrosynthesis through controlled voltage dosing and artificial intelligence. Proc. Natl. Acad. Sci. U. S. A..

[cit67] Casebolt R., Levine K., Suntivich J., Hanrath T. (2021). Pulse check: Potential opportunities in pulsed electrochemical CO2 reduction. Joule.

[cit68] Gupta N., Gattrell M., MacDougall B. (2006). Calculation for the cathode surface concentrations in the electrochemical reduction of CO2 in KHCO3 solutions. J. Appl. Electrochem..

