# Supplementary material for: Pulsed electrolysis: enhancing primary benzylic C(sp3)–H nucleophilic fluorination
Source: Org Chem Front. 2023 Dec 13;11(3):802–8. doi: 10.1039/d3qo01865b (PMC10825853; doi:10.1039/d3qo01865b)
Supplement: QO-011-D3QO01865B-s001 [file QO-011-D3QO01865B-s001.pdf]

Electronic Supplementary Material (ESI) for Organic Chemistry Frontiers.
This journal is © the Partner Organisations 2023

Supporting Information

Pulsed Electrolysis: Enhancing Primary Benzylic C(sp^3)–H Nucleophilic Fluorination

Alexander P. Atkins[a], Atul K. Chaturvedi[a], Joseph A. Tate[b], Alastair J. J. Lennox*[a]

[a]School of Chemistry, University of Bristol, Cantock's Close, BS81TS, Bristol United Kingdom

[b]Jealott's Hill International Research Centre, Syngenta, Bracknell, RG426ET, United Kingdom

*Corresponding Author; Alastair J. J. Lennox, a.lennox@bristol.ac.uk

Table of Contents

Supporting Information 1

Table of Contents 2

1. General Experimental Details 3

2. Cyclic Voltammograms 4

3. Reaction Optimisation 5

4. Control Reactions 11

5. Substrate Preparation and Characterisation 14

6. Product Preparation and Characterisation 18

7. Electrode Information 28

8. Mechanistic Rationale for Pulsed Electrolysis 30

9. Spectra of novel compounds 31

10. References 50

1. General Experimental Details

Air-sensitive procedures were carried out using Schlenk-line equipment under a N_2 atmosphere. Prior to use, glassware was dried at 180 °C overnight. Chemicals were purchased from Sigma Aldrich, Acros, Fluorochem, TCI, Lancaster, or Alfa Aesar and unless otherwise stated used without further purification. Additions of <200 µL were made with Gilson Pipetman pipettes. Anhydrous solvents were collected using an Anhydrous Engineering double alumina column drying system available at the University of Bristol. Anhydrous DCM was collected by distillation following refluxing over CaH_2 (5% w/v). rt = 25 °C. Technical grade solvents and silica gel (230-400 mesh, 60 Å pore size) were used for column chromatography or via a Biotage Selekt instrument with Sfär Silica D Duo capsules. Thin layer chromatography was performed on SiO_2 coated aluminium plates and visualized by ultraviolet fluorescence, potassium permanganate stain, or phosphomolybdic acid stain, or iodine vapours.

Analysis

NMR samples were submitted in $CDCl_3$ or MeCN-d_3 purchased from Sigma Aldrich and spectra were recorded on Brucker Nano 400, Jeol ECS 300, Jeol ECS 400 and Jeol ECZ 400, Bruker Advance III HD 500 cryo spectrometers. Chemical shifts are reported in parts per million (ppm). Coupling constants (*J*) are quoted in Hz. Multiplicities are reported as s (singlet), d (doublet), t (triplet), q (quartet), hept (heptet), m (multiplet). All yields are reported as ^{19}F NMR yields using 4,4-difluorobiphenyl or 1-fluoronapthalene as standards.

IR analysis was performed on a PerkinElmer Spectrum 100 FTIR with an ATR accessory and frequencies reported in wavenumbers (cm^{-1}).

High Resolution Mass Spectrometry (HRMS) was recorded on QExactive (GC-Orbitrap), Orbitrap Elite (LC-Orbitrap) or Synapt G2S (IMS-Q-TOF) instruments at the University of Bristol mass spectrometry facility. Samples were submitted in DCM, $CDCl_3$, MeCN or neat.

Electrochemical Considerations

Bulk Electrolysis experiments were performed using a PalmSens MultiPalmSens4 potentiostat. The MultiTrace software is capable of performing chromopotentiometry and chronoamperometry experiments. The use of multistep chronopotentiometry or chronoamperometry allows the construction of square-wave alternating polarity or pulsed electrolysis experiments. Platinum wire of 0.5 mm thickness was used to construct the electrodes and was purchased from Advent research materials (https://www.advent-rm.com/en-GB/Products/Pure-Metals/Platinum/Form/Wire/Line/PT5410).

Cyclic voltammetry analysis was performed using a PalmSens MultiSens4 potentiostat with platinum disk working electrodes, platinum wire electrodes and 0.1 M Ag/$AgNO_3$ reference electrodes. All electrodes were polished and the reaction mixtures stirred and degassed by a stream of N_2 gas for approximately 2 mins before each experiment. Each CV was then referenced to the Fc/Fc^+ redox couple by adding ferrocene to the reaction mixture and rerunning the CV.

HF solutions were used. These are extremely hazardous and therefore additional PPE was required. This included a double layer of long-cuff gloves to ensure no skin was exposed. A container of calcium gluconate gel was kept by the reaction in case of exposure. All needles containing HF were quenching in a bath of saturated aqueous $CaCO_3$ before disposal. Glass Schlenk tubes could be used repeatedly with Et_3N•3HF without any etching observed. Other amine•HF reagents (such as Et_3N•5HF, py•HF and mixtures of) resulted in etching of the glassware.

2. Cyclic Voltammograms

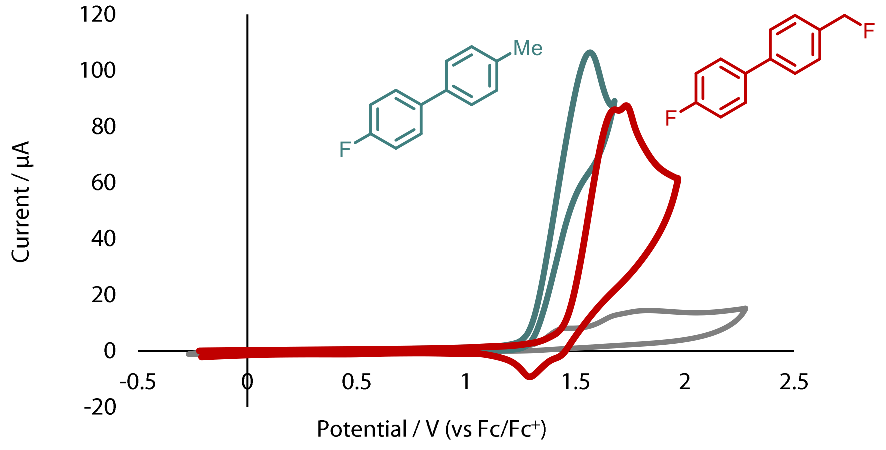

Figure S1. Cyclic voltammetry of the model substrate **1a** and model product **2a** vs Fc/Fc⁺

1a or **2a** (5 mM) in DCM (4 mL, anhydrous and degassed by a stream of N_2) and $TBAPF_6$ (0.1 M) as supporting electrolyte. Platinum disc working electrode, platinum wire counter electrode and Ag wire reference electrode referenced to Fc/Fc⁺ redox couple.

3. Reaction Optimisation

Table S1. Solvent screen

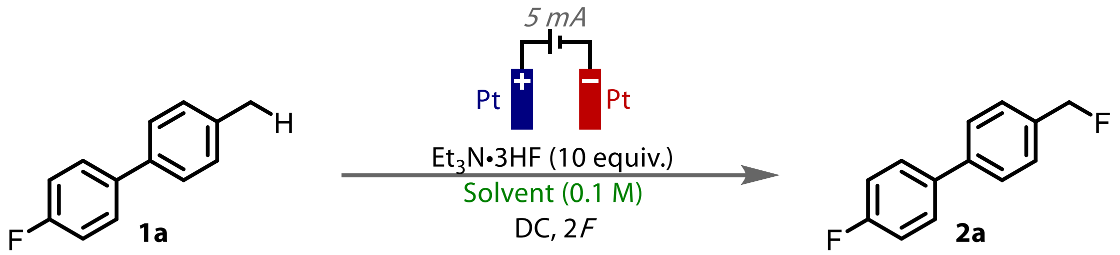

Entry	Solvent	2a Yield/ % [a]
1	DCM	34
2	$CHCl_3$	25
3	MeCN	10
4	DCM: MeCN (3:1)	8
5	DME	16
6	*t*-BuCN	1
7	DMF	0

[a] Yields determined by ^{19}F NMR using 4,4'-difluorobiphenyl as a standard.

Table S2. Anode materials screen

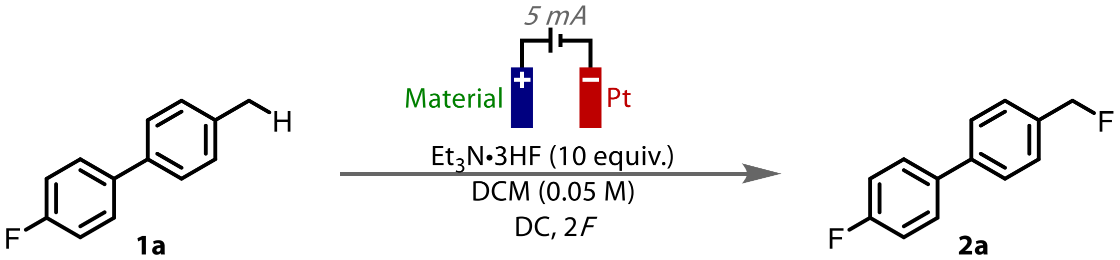

Entry	Anode Material	2a Yield/ % [a]
1	Platinum	34
2	BDD	21
3	RVC	14
4	Graphite	3
6	Nickel	0
7	Fluorine-doped tin Oxide	0

[a] Yields determined by ^{19}F NMR using 4,4'-difluorobiphenyl as a standard.

Platinum was selected as the counter electrode material due to it's low overpotential for proton reduction (the counter electrode process in this reaction).[1] However, it was found that other cathodic materials, such as allotropes of carbon or nickel foil, could be used without much impact on yield. However, platinum was chosen as the desired counter electrode material due to its inertness to HF•Amine mixtures.

Table S3. Alternating polarity screen

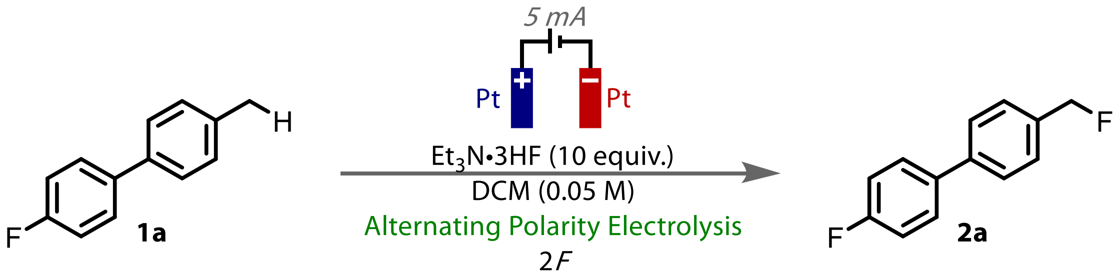

Entry	Period 1 / s	Period 2 / s	2a yield / %[a]	Deviation from above
1	180	10	38	
2	90	10	44	
3	90	10	33	2 mA current
4	60	60	30	
5	60	10	40	
6	30	5	40	
7	5	5	14	
8	5	5	38	10 mA current
9	0.2	0.2	0	
10	0.2	0.2	8	20 mA current
11	0.2	0.2	12	30 mA current
12	0.1	0.1	10	20 mA current

[a] Yields determined by ^{19}F NMR using 1-fluoronaphthalene or 4,4'-difluoro-1,1'-biphenyl as a standard.

Table S4. Step Potential Screen

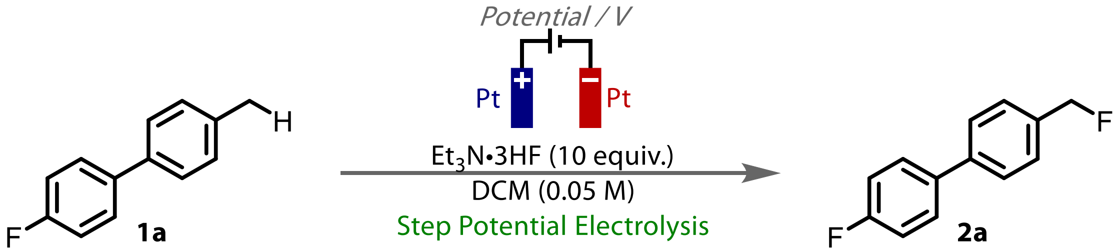

Entry	S_1 / V[b]	S_1 / s	S_2 / V[b]	S_2 / s	Charge Passed / *F*	2a yield / %[a]
1	1.06	30	-0.24	5	0.2	0
2	1.36	30	-0.24	5	2.3	53
3[c]	1.36	30	-0.24	5	1.7	49
4	1.36	30	-0.74	5	1.5	27
5	1.56	30	-0.24	5	2.6	39 [d]

[a] Yields determined by ^{19}F NMR using 1-fluoronaphthalene or 4,4'-difluoro-1,1'-biphenyl as a standard. [b] Potentials vs Fc/Fc$^+$. [c] with 0.1 M $TBAPF_6$ additional supporting electrolyte. [d] 3% difluorination observed

Table S5. Effect of variation of t_{on} vs yield results

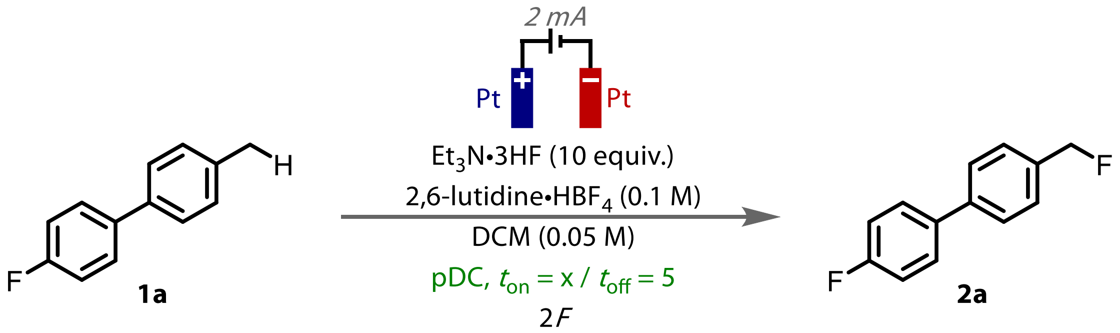

Entry	t_{on} / s	2a yield / %[a]
1	5	37
2	15	51
3	30	57
4	60	40
5	90	42

[a] Yields determined by ^{19}F NMR using 1-fluoronaphthalene as a standard.

Table S6. Effect of variation of t_{off} vs yield results

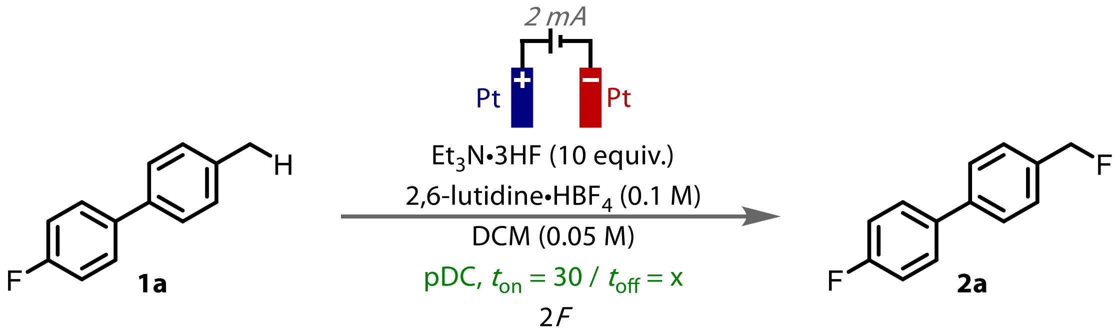

Entry	t_{off} / s	2a yield / % [a]
1	0	43
2	0.5	53
3	2.5	53
4	5	57
5	10	48
6	20	47
7	30	54
8	60	50

[a] Yields determined by ^{19}F NMR using 1-fluoronaphthalene as a standard.

Table S7. Effect of $Et_3N{\bullet}3HF$ loading on reaction outcome

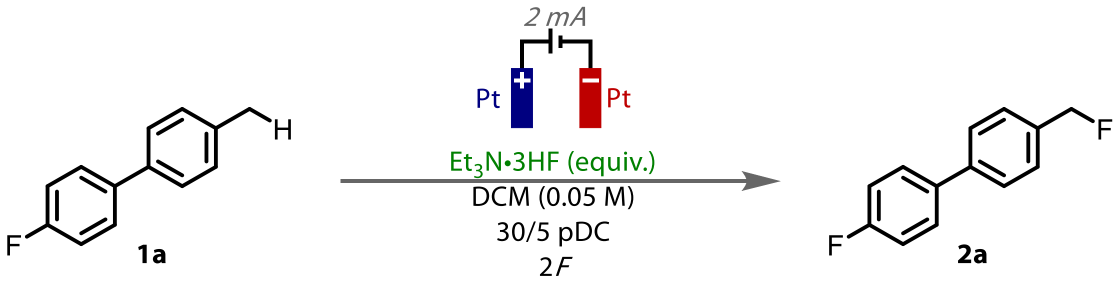

Entry	$Et_3N{\bullet}3HF$ / equiv.	2a Yield/ % [a]
1	2	41
2	4	47
3	10	53
4	20	47
5	50	50

[a] Yields determined by ^{19}F NMR using 4,4’-difluorobiphenyl or 1-fluoronaphthalene as a standard.

Table S8. Current screen

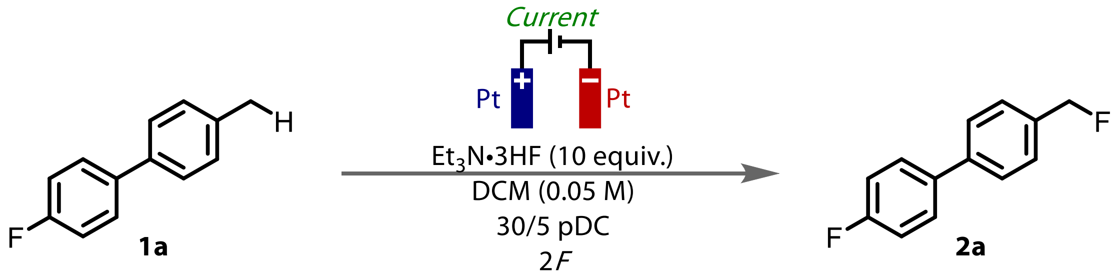

Entry	Current / mA	2a yield / % [a]
1	1	33
2	2	53
3	3	43
4	5	50

[a] Yields determined by ^{19}F NMR using 4,4’-difluorobiphenyl as a standard.

Table S9. Concentration screen

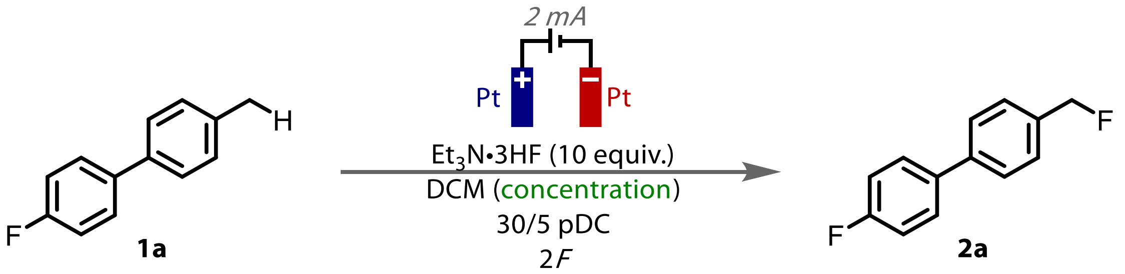

Entry	Concentration / M	2a yield / % [a]
1	0.10	25
2	0.05	53
3	0.03	35
4	0.02	37
5	0.01	29

[a] Yields determined by ^{19}F NMR using 4,4'-difluorobiphenyl or 1-fluoronaphthalene as a standard.

Table S10. Stirring rate screen

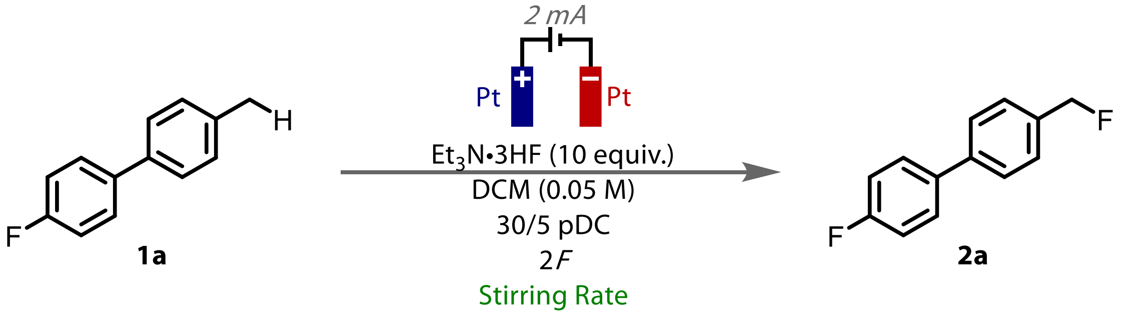

Entry	Stirring Rate / rpm	2a yield / % [a]
1	0	33
2	250	42
3	500	49
4	1000	53
5	1500	50

[a] Yields determined by ^{19}F NMR using 4,4'-difluorobiphenyl or 1-fluoronaphthalene as a standard.

Unless otherwise stated, all reactions during the optimisation process were run at 1000 rpm.

Table S11. Additional supporting electrolyte screen

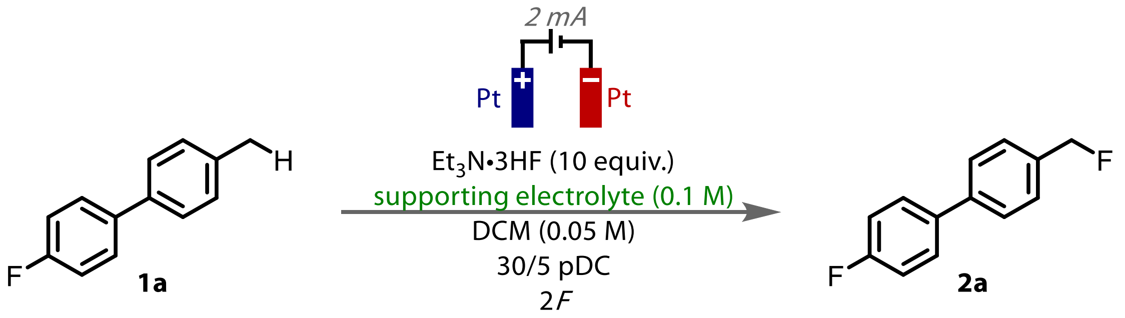

Entry	Supporting Electrolyte	2a yield/ % [a]
1	None	53
2	$TBAPF_6$	48
3	$TBABF_4$	51
4	$THexPF_6$	41
5	*n*$PrNPF_6$	37
6	$TEABF_4$	43
7	$TEAPF_6$	40
8	2,6-lutidine•HBF_4	57
9[b]	2,4,6-collidine•HBF_4	55
10[b]	Et_3N•HBF_4	51

[a] Yields determined by ^{19}F NMR using 1-fluoronaphthalene or 4,4'-difluorobiphenyl as a standard. [b] 0.4 M additional supporting electrolyte concentration.

4. Control Reactions

BF_4^- as a fluoride source control

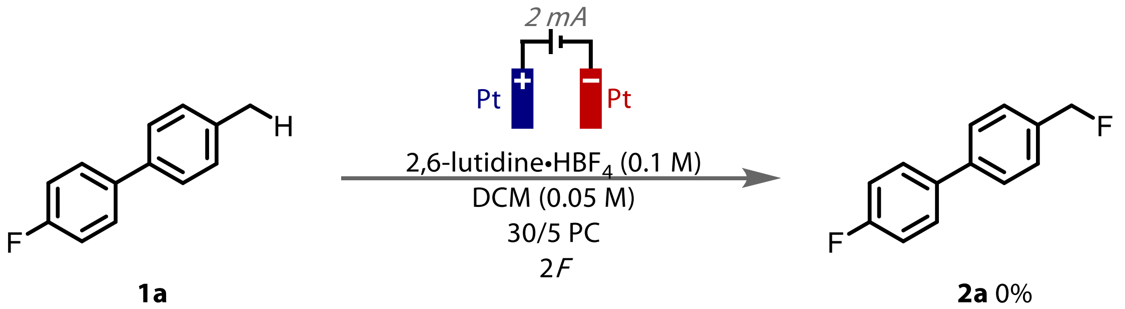

Scheme S1. BF_4 salt as a fluoride source control reaction.

Under the standard reaction conditions but in the absence of Et_3N•3HF no benzyl fluoride **2a** was observed indicating that the BF_4 salt is not a competent fluoride source for capturing primary benzylic cations.

Oxidation of Product

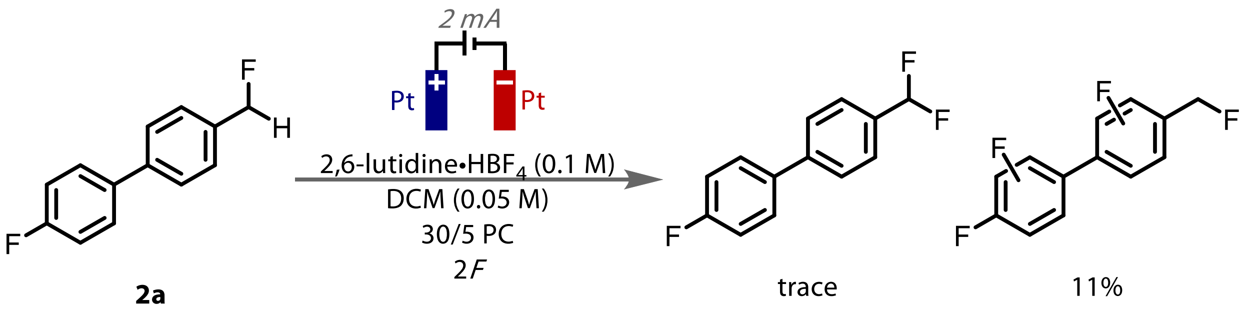

Scheme S2. Subjecting **2a** to the reaction conditions.Yields calculated vs 1-fluoronaphthalene ^{19}F NMR standard.

Subjecting model product **2a** to the reaction conditions results in trace amounts of difluorinated product. 1% **2a** remains after passage of 2 *F* of current. 11% of suspected ring-fluorinated products is observed. This suggests that oxidation of **2a** is unproductive and leads to decomposition (explained by the low mass balance observed in this control reaction).

Constant Potential vs Step Pulsed Constant Potential on Product Formation

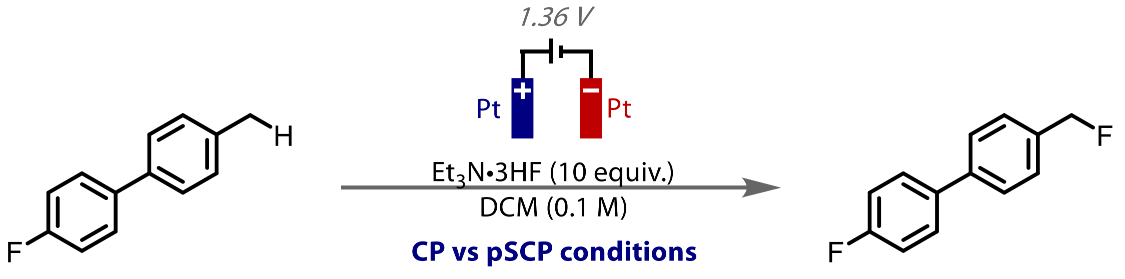

1a **2a**

The direct potential vs step potential control reaction was carried out on a 0.5 mmol scale at increased concentration (0.1 M instead of 0.05 M). 0.25 mmol of 4,4-difluorobiphenyl was added as an internal standard. Electrolysis was carried out at 1.36 V (vs Fc/Fc^{+}) for alternating periods of 45 mins of applied potential, first under constant potential (CP) conditions, then pulsed step constant potential (SP) conditions (90 cycles of S_1 = 30 sec V = 1.36; S_2 = 5 sec V = -0.24). Aliquots were taken at the end of each cycle.

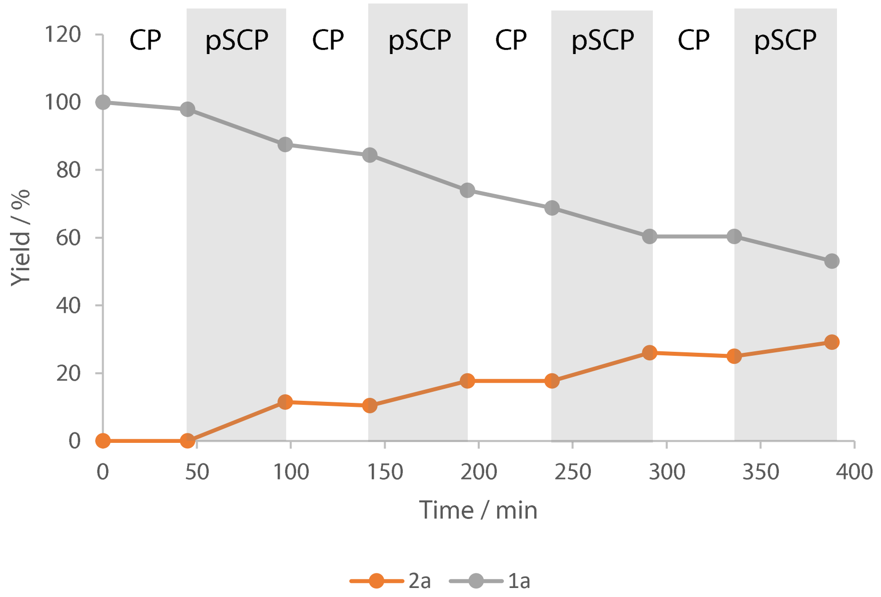

Figure S2. On/off experiment alternating between CP and pSCP electrolysis showing loss of starting material (**1a** – grey line) and increase in product (**2a**).

Pulsed Constant Potential Control

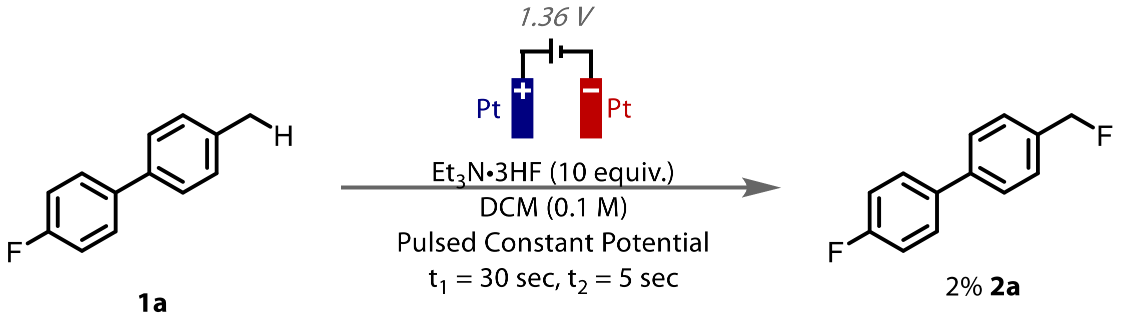

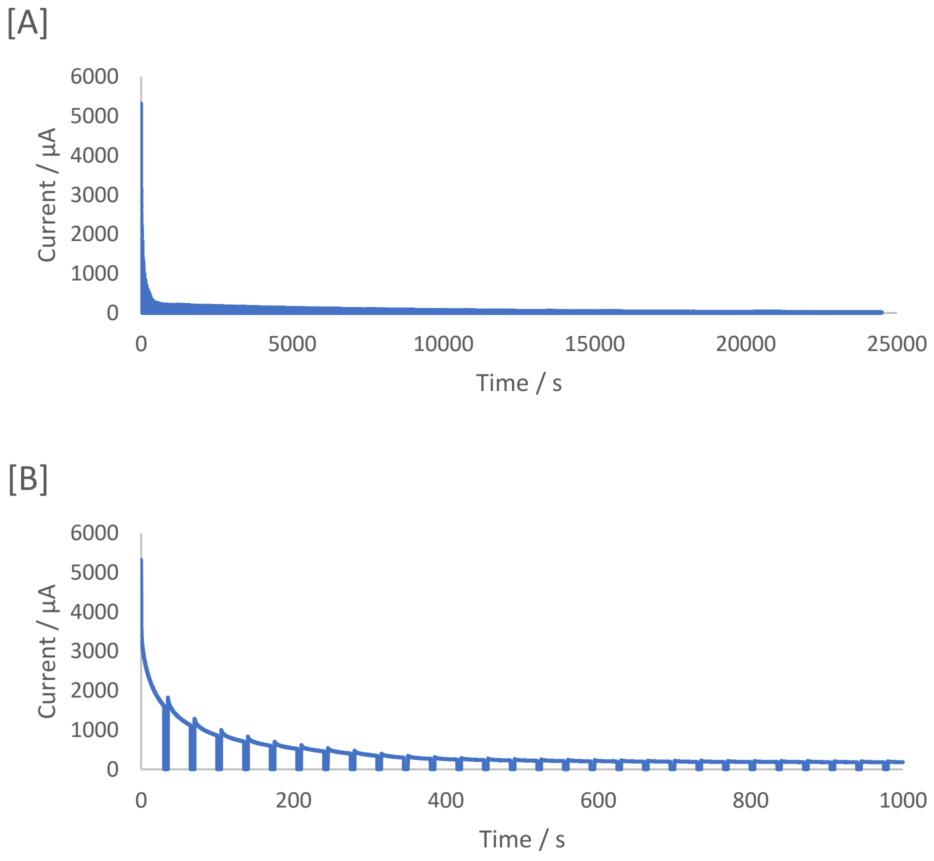

Figure S3. Chronoamperometry trace of pulsed potential control experiment. [A] Shows the full trace over 250000 seconds. [B] Shows the trace for the first 1000 seconds to illustrate the finer shape of the curve.

A pulsed constant potential (pCP) control experiment was conducted using the optimal pulse sequence (t_{on} = 30 sec, t_{off} = 5 sec) at 1.36 V (vs Fc/Fc^+). The overall result followed that of the constant potential experiment, trending towards unworkably low currents. Only 2% **2a** was observed in the crude ^{19}F NMR.

5. Substrate Preparation and Characterisation

General Procedure 1: Preparation of biphenyl starting materials

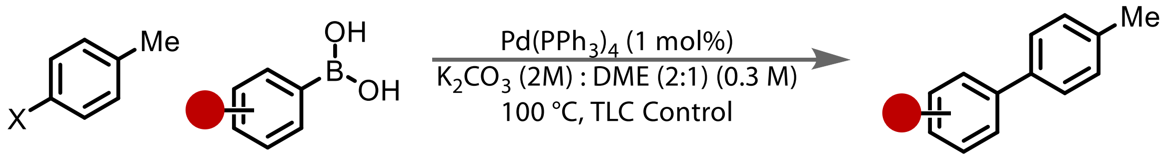

To a round-bottom flask equipped with a stirrer bar was added boronic acid (1.05 equiv.), aryl halide (1 equiv.) and $Pd(PPh_3)_4$ (0.01 equiv.). To this was added 2:1 DME and 2M aq. K_2CO_3 solution (0.3 M with respect to aryl halide) and the reaction mixture was stirred in a pre-heated heating mantle set to 100 °C. Reaction progress was monitored by TLC. Upon complete consumption of the starting material the reaction was cooled to rt and quenched with sat. aq. NH_4Cl. The resulting mixture was partitioned and organics extracted into DCM (3x 30 mL). The organic extracts were combined, dried with $MgSO_4$, filtered, and dried under reduced pressure to afford crude product, which was purified by column chromatography to afford pure product.

4-Fluoro-4'-methyl-1,1'-biphenyl **1a**

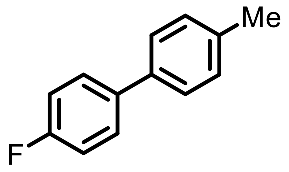

4-Fluoro-4'-methyl-1,1'-biphenyl was prepared from 4-iodotoluene and 4-fluorophenylboronic acid via general procedure 1 on a 10.0 mmol scale and purified by column chromatography (100% pentane) to afford a white solid (1830 mg, 98%).

^{1}H NMR: (400 MHz, $CDCl_3$) δ 7.56 – 7.50 (m, 2H), 7.46 – 7.42 (m, 2H), 7.26 – 7.22 (m, 2H), 7.15 – 7.08 (m, 2H), 2.39 (s, 3H).
^{19}F NMR: (376 MHz, $CDCl_3$) δ -116.2 (tt, *J* = 8.7, 5.4 Hz).
^{13}C NMR: (101 MHz, $CDCl_3$) δ 162.4 (d, J = 245.8 Hz), 137.5, 137.4 (d, J = 3.1 Hz), 137.2, 129.7, 128.6 (d, J = 7.9 Hz), 127.0, 115.7 (d, J = 21.4 Hz), 21.2.
Spectroscopic data are in agreement with the literature.[2]

4-Bromo-4'-methyl-1,1'-biphenyl **1c**

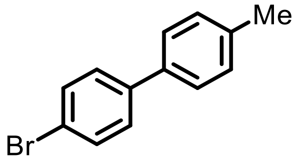

4-Bromo-4′-methyl-1,1′-biphenyl was prepared from 4-bromo-iodobenzene and 4-methylphenylboronic acid via general procedure 1 on a 5.0 mmol scale and purified by column chromatography (100% pentane) to afford a white solid (964 mg, 78%).

^{1}H NMR (400 MHz, $CDCl_3$) δ 7.57 (d, *J* = 8.2 Hz, 1H), 7.48 (dd, *J* = 8.1, 5.6 Hz, 2H), 7.28 (d, *J* = 7.7 Hz, 1H), 2.43 (s, 3H).
^{13}C NMR (101 MHz, $CDCl_3$) δ 140.2, 137.6, 137.2, 131.9, 129.7, 128.7, 126.9, 121.3, 21.2.
Data are in agreement with the literature.[3]

4-Methyl-4′-(trifluoromethyl)-1,1′-biphenyl **1d**

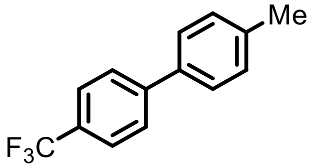

4-Methyl-4′-(trifluoromethyl)-1,1′-biphenyl was prepared from 4-iodotoluene and 4-trifluomethylphenylboronic acid via general procedure 1 on a 2.5 mmol scale and purified by column chromatography (100% pentane) to afford a white solid (569.1 mg, 95%).

^{1}H NMR: (400 MHz, $CDCl_3$) δ 7.68 (s, 4H), 7.50 (d, J = 8.2 Hz, 2H), 7.28 (d, *J* = 7.9 Hz, 2H), 2.41 (s, 3H).
^{19}F NMR: (376 MHz, $CDCl_3$) δ -62.2 (s, 3H).
^{13}C NMR: (101 MHz, $CDCl_3$) δ 144.8, 138.3, 137.0, 129.9, 129.2 (d, J = 32.1 Hz), 127.3, 127.3, 125.8 (d, *J* = 3.6 Hz), 123.2 (q, *J* = 272.8 Hz), 21.3.
Spectroscopic data are in agreement with the literature.[4]

4-Chloro-4'-methyl-1,1'-biphenyl **1e**

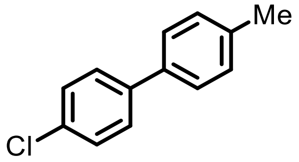

4-Chloro-4′-methyl-1,1′-biphenyl was prepared from (4-chlorophenyl)boronic acid and 4-iodotoluene via general procedure 1 on a 5.0 mmol scale and purified by column chromatography (100% pentane) to afford a white solid (831 mg, 82%).

^{1}H NMR (400 MHz, $CDCl_3$) δ 7.53 – 7.49 (m, 2H), 7.48 – 7.44 (m, 2H), 7.42 – 7.38 (m, 2H), 7.26 (d, *J* = 7.9 Hz, 2H), 2.41 (s, 3H).
^{13}C NMR (101 MHz, $CDCl_3$) δ 139.7, 137.6, 137.3, 133.2, 129.7, 129.0, 128.3, 127.0, 21.2.
Data are in agreement with the literature.[5]

3'-Bromo-5'-fluoro-2-methyl-1,1'-biphenyl ***1f***

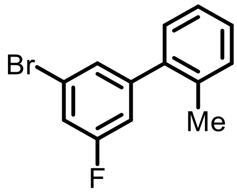

3’bromo-5’-fluoro-2 -methyl-1,1’-biphenyl was prepared from 1-bromo-3-fluoro-5-iodobenzene and 2-methylphenylboronic acid via general procedure 1 on a 5.0 mmol scale and purified by column chromatography (100% pentane) to afford a colourless liquid (mass 1113 mg, 84%).

^{1}H NMR (400 MHz, $CDCl_3$) δ 7.35 – 7.24 (m, 5H), 7.21 (dd, *J* = 7.3, 1.3 Hz, 1H), 7.01 (ddd, *J* = 9.2, 2.4, 1.4 Hz, 1H), 2.30 (s, 3H).
^{19}F NMR (377 MHz, $CDCl_3$) δ -111.07 – -111.41 (m).
^{13}C NMR (101 MHz, $CDCl_3$) δ 162.4 (d, *J* = 250.6 Hz), 145.6 (d, *J* = 8.3 Hz), 139.4 (d, *J* = 1.9 Hz), 135.3, 130.7, 129.6, 128.3 (d, *J* = 3.0 Hz), 128.3, 126.1, 122.3 (d, *J* = 10.2 Hz), 117.6 (d, *J* = 24.4 Hz), 115.4 (d, *J* = 21.3 Hz), 20.4.
HRMS (EI$^+$) calc: [M$^+$] ($C_{13}H_{10}BrF$) 263.9944; measured: 263.9942 = 0.76 ppm error
IR (neat) vmax/cm^{-1}: 2972, 2901, 1601, 1581, 1567, 1417, 1178, 1078, , 1066, 909, 757.

4-Bromo-2-chloro-4'-methyl-1,1'-biphenyl ***1g***

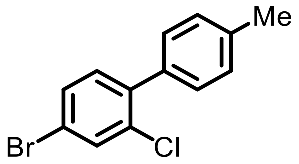

4-Bromo-2-chloro-4’-methyl-1,1’-biphenyl was prepared from 4-bromo-2-chloro-1-iodobenzene and 4-methylphenylboronic acid via general procedure 1 on a 5.0 mmol scale and purified by column chromatography (100% pentane) to afford a white solid (957 mg, 68%).

^{1}H NMR (400 MHz, $CDCl_3$) δ 7.66 (d, *J* = 2.0 Hz, 1H), 7.46 (dd, *J* = 8.2, 2.0 Hz, 1H), 7.35 – 7.31 (m, 2H), 7.29 – 7.25 (m, 2H), 7.22 (d, *J* = 8.2 Hz, 1H), 2.43 (s, 3H).
^{13}C NMR (101 MHz, $CDCl_3$) δ 139.7, 137.9, 135.6, 133.7, 132.6, 132.6, 130.2, 129.3, 129.0, 121.3, 21.4.
HRMS (EI$^+$) calc: [M$^+$] ($C_{13}H_{10}BrCl$) 279.9649; measured: 279.9646 = 1.07 ppm error
IR (neat) vmax/cm^{-1}: 3726, 3624, 2972, 2920, 1581, 1466, 1871, 1084, 1072, 810.

Synthesis of methyl (4'-methyl-[1,1'-biphenyl]-4-yl) carbonate 1b

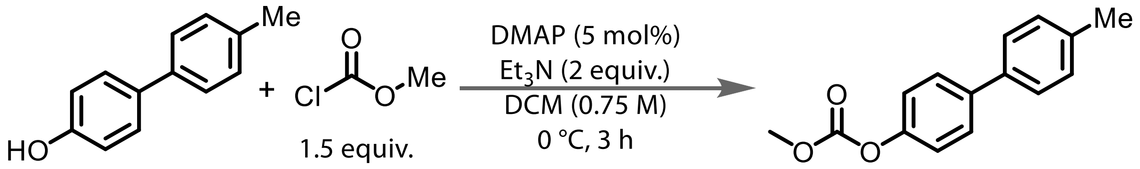

To an oven dried Schlenk containing a magnetic stirrer bar and under an atmosphere of nitrogen was added 4'-methyl-[1,1'-biphenyl]-4-ol (400 mg, 2.2 mmol, 1.0 equiv.) and DMAP (13.5 mg, 0.1 mmol, 0.05 equiv.) which was then dissolved in DCM (3 mL) and Et_3N (604 µL, 4.3 mmol, 2 equiv.). The reaction mixture was then cooled to 0 °C and methyl chloroformate (251 µL, 3.3 mmol, 1.5 equiv.) was added slowly. The reaction mixture was stirred and allowed to warm to room temperature slowly. After 5 hours the reaction was quenched with DI H_2O (5 mL). The resulting mixture was extracted into DCM (3x20 mL), dried with $MgSO_4$, filtered, then dried under vacuum. The remaining crude mixture was purified by column chromatography (10% ethyl acetate in pentane) to afford the product as a white solid (292 mg, 56%).

^{1}H NMR (400 MHz, $CDCl_3$) δ 7.62 – 7.56 (m, 2H), 7.50 – 7.46 (m, 2H), 7.29 – 7.22 (m, 4H), 3.94 (s, 3H), 2.41 (s, 3H).
^{13}C NMR (101 MHz, $CDCl_3$) δ 154.5, 150.4, 139.3, 137.5, 137.4, 129.7, 128.1, 127.1, 121.4, 55.6, 21.2.
HRMS (EI$^+$) calc: [M$^+$] ($C_{15}H_{14}O_3$) 242.0937; measured: 242.0939 = 0.8 ppm error
IR (neat) vmax/cm^{-1}: 3040, 3030, 2972, 2917, 2867, 1755, 1498, 144, 1252, 1227, 1210, 933, 809, 776, 493

6. Product Preparation and Characterisation

General Procedure 2: Preparation of primary benzyl fluorides

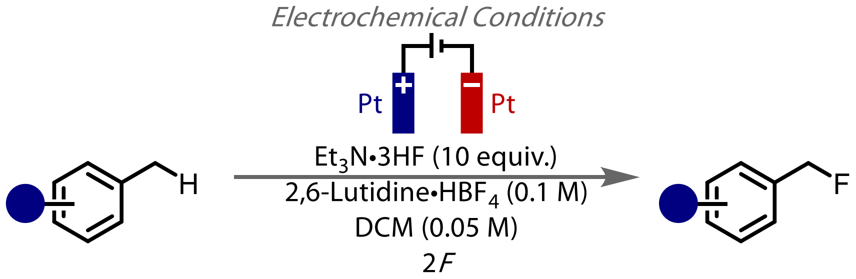

To an oven-dried 10 mL Young's tube equipped with a stirrer bar was added substrate (0.2 mmol, 1 equiv.) and 2,6-lutidine•HBF_4 (0.4 mmol, 2 equiv., 77.78 mg). The vessel was evacuated and back-filled with N_2 three times. DCM (4 mL) was added and the reaction mixture allowed to stir for 5 min. To this was added Et_3N•3HF (2.0 mmol, 10 equiv., 0.33 mL). The seal was replaced by a Suba-seal installed with a platinum anode, a platinum cathode and a silver wire reference electrode. The reaction mixture was stirred and subjected to electrolysis (see below). Upon completion, the electrodes were removed and sat. aq. $NaHCO_3$ (6 mL) was added. The aqueous layer was stirred until basic (determined by pH strips). The reaction mixture was partitioned and diluted with DCM (10 mL), and H_2O (10 mL) and brine (10 mL) were added. The crude mixture was extracted into DCM (2 x 10 mL). Solvent was then removed under reduced pressure. Yield was determined by ^{19}F NMR using 1-fluoronaphthalene or 4,4'-difluorobiphenyl as standards. Crude mixtures were combined and purified by column chromatography (EtOAc in pentane) to afford pure products.

Electrolysis conditions:

DC electrolysis: 2 mA constant current for 2 *F*.

pDC electrolysis: t_{on} = 30 seconds (2 mA), t_{off} = 5 seconds (0 mA) for 2 *F*.

Each substrate was subjected to the DC and pDC conditions 3 times and NMR yields recorded. The desired benzyl fluoride product was then isolated to confirm its identity.

The average standard deviation for the pDC experiments was 3.53. The average standard deviation for the DC experiments was 3.23.

4-Fluoro-4'(fluoromethyl)-1,1'-biphenyl **2a**

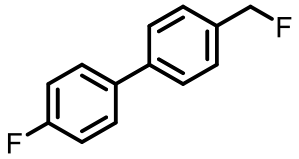

4-Fluoro-4'-(fluoromethyl)-1,1'-biphenyl was prepared from 4-fluoro-4'methyl-1,1'-biphenyl via general procedure 2 and purified via column chromatography (100% pentane) to afford title product as a white solid.

^{1}H NMR (400 MHz, $CDCl_3$) δ 7.60 – 7.52 (m, 4H), 7.48 – 7.43 (m, 2H), 7.17 – 7.10 (m, 2H), 5.42 (d, *J* = 47.8 Hz, 2H).
^{19}F NMR (377 MHz, $CDCl_3$) δ -115.2 (tt, *J* = 8.6, 5.3 Hz), -206.4 (t, *J* = 47.8 Hz).
^{13}C NMR (101 MHz, $CDCl_3$) δ 162.7 (d, *J* = 246.7 Hz), 140.9 (d, *J* = 3.1 Hz), 136.8 (d, *J* = 2.5 Hz), 135.3 (d, *J* = 17.1 Hz), 128.9 (d, *J* = 8.1 Hz), 128.3 (d, *J* = 5.7 Hz), 127.4, 115.9 (d, *J* = 21.5 Hz), 84.5(d, *J* = 166.1 Hz).
HRMS (EI$^+$) calc: [M$^+$] ($C_{13}H_{10}F_2$) 204.0745; measured: 204.0736 = 4.4 ppm error
IR (neat) vmax/cm^{-1}: 2991, 2926, 2856, 1526, 1605, 1500, 1378, 1247, 1161, 982, 818, 802, 734

Competition Experiment Results

Run	**2a** / % (pDC)	**2a** / % (DC)
1	57	38
2	56	43
3	56	42
Average	56.3	41
Standard Deviation	0.58	2.65

4'-(Fluoromethyl)-[1,1'-biphenyl]-4-yl methyl carbonate ***2b***

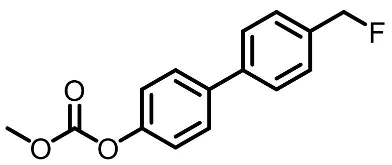

4'-(fluoromethyl)-[1,1'-biphenyl]-4-yl methyl carbonate was prepared from methyl (4'-methyl-[1,1'-biphenyl]-4-yl) carbonate via general procedure 2 and purified by column chromatography (10% ethyl acetate in pentane) to afford the title product as a white solid. Isolated with trace amounts of the difluorinated product.

^{1}H NMR (400 MHz, $CDCl_3$) δ 7.63 – 7.58 (m, 4H), 7.49 – 7.43 (m, 2H), 7.32 – 7.23 (m, 2H), 5.43 (d, *J* = 47.8 Hz, 2H), 3.94 (s, 3H).
^{19}F NMR (376 MHz, $CDCl_3$) δ -206.5 (t, *J* = 47.8 Hz).
^{13}C NMR (151 MHz, $CDCl_3$) δ 154.4, 150.9, 140.9 (d, *J* = 3.1 Hz), 138.8, 135.5 (d, *J* = 17.1 Hz), 128.4, 128.2 (d, *J* = 5.7 Hz), 127.5, 121.5, 84.5 (d, *J* = 166.2 Hz), 55.6.
HRMS (EI$^+$) calc: [M$^+$] ($C_{15}H_{13}O_3F$) 260.0843; measured: 260.0847 = 1.5 ppm error
IR (neat) vmax/cm^{-1} 3049, 2961, 2899, 2861, 1762, 1501, 1443, 1297, 1262, 1232, 936, 809, 732

Competition Experiment Results

Run	**2b** / % (pDC)	**2b** / % (DC)
1	46	33
2	56	34
3	43	33
Average	48.3	33.3
Standard Deviation	6.81	0.58

4-Bromo-4'-(fluoromethyl)-1,1'-biphenyl ***2c***

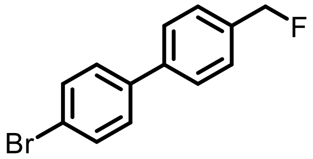

4-Bromo-4'-(fluoromethyl)-1,1'-biphenyl was prepared from 4-bromo-4'-methyl-1,1'-biphenyl via general procedure 2 and purified by column chromatography (100% pentane) to afford the title product as a white solid.

^{1}H NMR (400 MHz, $CDCl_3$) δ 7.60 – 7.55 (m, 4H), 7.48 – 7.44 (m, 4H), 5.43 (d, *J* = 47.8 Hz, 2H).
^{19}F NMR (377 MHz, $CDCl_3$) δ -206.97 (t, *J* = 47.7 Hz).
^{13}C NMR (101 MHz, $CDCl_3$) δ 140.6 (d, *J* = 3.2 Hz), 139.6, 135.7 (d, *J* = 17.3 Hz), 132.1, 128.9, 128.2 (d, *J* = 5.8 Hz), 127.3 (d, *J* = 1.5 Hz), 122.0, 84.4 (d, *J* = 166.5 Hz).
HRMS (EI$^+$) calc: [M$^+$]($C_{13}H_{10}FBr$) 263.9944, measured 263.9944, 0.00 ppm error
IR (neat) vmax/cm^{-1} 2995, 2852, 2329, 2157, 1904, 1587, 1482, 1388, 1078, 1002

Competition Experiment Results

Run	**2c** / % (pDC)	**2c** / % (DC)
1	63	41
2	61	53
3	59	50
Average	61	48
Standard Deviation	2.00	6.24

4-(Fluoromethyl)-4'-(trifluoromethyl)-1,1'-biphenyl **2d**

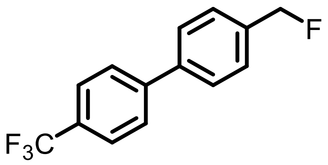

4-(Fluoromethyl)-4'-(trifluoromethyl)-1,1'-biphenyl was prepared from 4-methyl-4'-(trifluoromethyl)-1,1'-biphenyl via general procedure 2 and purified by column chromatography (100% pentane) to afford the title product as a white solid.

^{1}H NMR (400 MHz, $CDCl_3$) δ 7.70 (s, 4H), 7.66 – 7.61 (m, 2H), 7.53 – 7.46 (m, 2H), 5.45 (d, *J* = 47.7 Hz, 2H).
^{19}F NMR (377 MHz, $CDCl_3$) δ -62.5 (s, 3F), -207.7 (t, *J* = 47.7 Hz, 1F).
^{13}C NMR (151 MHz, $CDCl_3$) δ 144.3, 140.3 (d, *J* = 3.1 Hz), 136.3 (d, *J* = 17.3 Hz), 129.8 (q, *J* = 32.4 Hz), 128.2 (d, *J* = 6.1 Hz), 127.7 (d, *J* = 1.3 Hz), 127.6, 125.9 (q, *J* = 3.8 Hz), 124.4 (q, *J* = 272.3 Hz), 84.3 (d, *J* = 166.4 Hz).
HRMS (EI^{+}) calc: [M^{+}] ($C_{14}H_{10}F_4$) 254.0713; measured: 254.0708 = 1.97 ppm error
IR (neat) vmax/cm^{-1}: 2925, 1615, 1326, 1275, 1171, 1128, 1015, 909, 813, 737, 620.

Competition Experiment Results

Run	**2d** / % (pDC)	**2d** / % (DC)
1	30	21
2	31	24
3	36	29
Average	32.3	24.7
Standard Deviation	3.21	4.04

4-Chloro-4'-(fluoromethyl)-1,1'-biphenyl **2e**

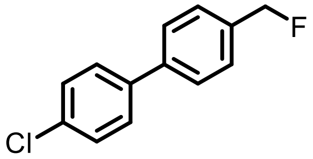

3'-Bromo-5'-fluoro-2-(fluoromethyl)-1,1'-biphenyl was prepared from 4-chloro-4'-methyl-1,1'-biphenyl via general procedure 2 and purified by column chromatography (100% pentane) to afford the title product as a white solid. Isolated with trace amounts of the difluorinated product.

^{1}H NMR (400 MHz, $CDCl_3$) δ 7.61 – 7.56 (m, 2H), 7.55 – 7.50 (m, 2H), 7.49 – 7.40 (m, 4H), 5.43 (d, *J* = 47.8 Hz, 2H).
^{19}F NMR (377 MHz, $CDCl_3$) δ -206.9 (t, *J* = 47.9 Hz).
^{13}C NMR (101 MHz, $CDCl_3$) δ 140.6 (d, *J* = 3.2 Hz), 139.2, 135.7 (d, *J* = 17.0 Hz), 133.8, 129.1, 128.5, 128.2 (d, *J* = 5.8 Hz), 127.3 (d, *J* = 1.5 Hz), 84.4 (d, *J* = 166.3 Hz).
HRMS (EI$^+$) calc: [M^+]($C_{13}H_{10}ClF$) 220.0450, measured 220.0448, 0.91 ppm error
IR (neat) vmax/cm^{-1} 3029, 2852, 1702, 1606, 1484, 1392, 1364, 1095, 1014, 1004, 807

Competition Experiment Results

Run	**2e** / % (pDC)	**2e** / % (DC)
1	44	36
2	45	38
3	37	32
Average	42	35.3
Standard Deviation	4.36	3.06

3'-Bromo-5'-fluoro-2-(fluoromethyl)-1,1'-biphenyl ***2f***

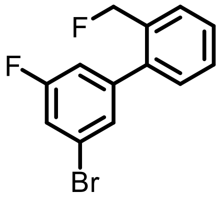

3'-Bromo-5'-fluoro-2-(fluoromethyl)-1,1'-biphenyl was prepared from 3'-bromo-5'-fluoro-2-methyl-1,1'-biphenyl via general procedure 2 and purified by column chromatography (100% pentane) to afford the title product as a sticky yellow liquid.

^{1}H NMR (400 MHz, $CDCl_3$) δ 7.60 – 7.53 (m, 1H), 7.50 – 7.41 (m, 2H), 7.35 – 7.26 (m, 3H), 7.06 (ddd, *J* = 9.2, 2.4, 1.4 Hz, 1H), 5.27 (d, *J* = 48.0 Hz, 2H).
^{19}F NMR (377 MHz, $CDCl_3$) δ -110.6 – -110.7 (m), -198.7 (t, *J* = 47.9 Hz).
^{13}C NMR (101 MHz, $CDCl_3$) δ 162.4 (d, *J* = 251.4 Hz), 143.6 (d, *J* = 8.3 Hz), 139.9, 133.3 (d, *J* = 15.9 Hz), 130.4 (d, *J* = 6.4 Hz), 130.0 (d, *J* = 2.0 Hz), 129.5 (d, *J* = 3.6 Hz), 128.8 (d, *J* = 2.0 Hz), 128.5 – 128.3 (m), 122.6 (d, *J* = 10.1 Hz), 118.3 (d, *J* = 24.4 Hz), 115.6 (dd, *J* = 21.7, 2.4 Hz), 83.4 (d, *J* = 166.1 Hz).
HRMS (EI^{+}) calc: [M^{+}]($C_{13}H_9BrF_2$) 281.9850, measured 281.9848, 0.71 ppm error
IR (neat) νmax/cm^{-1} 2960, 2923, 2852, 1601, 1568, 1418, 1180, 1000, 976, 910.

Competition Experiment Results

Run	**2f** / % (pDC)	**2f** / % (DC)
1	30	20
2	32	24
3	27	23
Average	29.7	22.3
Standard Deviation	2.52	2.08

*4-Bromo-2-chloro-4'-(fluoromethyl)-1,1'-biphenyl **2g***

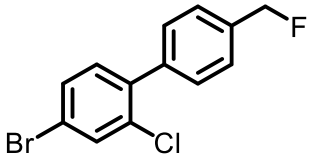

4-bromo-2-chloro-4'-(fluoromethyl)-1,1'-biphenyl was prepared from 4-bromo-2-chloro-4'-methyl-1,1'-biphenyl via general procedure 2 and purified by column chromatography (100% pentane) to afford the title product as a colourless solid.

^{1}H NMR (400 MHz, $CDCl_3$) δ 7.68 (d, *J* = 2.0 Hz, 1H), 7.48 – 7.45 (m, 4H), 7.28 (s, 1H), 7.23 (d, *J* = 8.2 Hz, 1H), 5.46 (d, *J* = 47.7 Hz, 2H).
^{19}F NMR (377 MHz, $CDCl_3$) δ -208.0 (t, *J* = 47.7 Hz).
^{13}C NMR (101 MHz, $CDCl_3$) δ 139.1, 138.9 (d, *J* = 3.0 Hz), 136.1 (d, *J* = 17.3 Hz), 133.6, 132.7, 132.5, 130.3, 129.7, 127.4 (d, *J* = 5.9 Hz), 121.8, 84.4 (d, *J* = 166.7 Hz).
HRMS (EI$^+$) calc: [M$^+$]($C_{13}H_9FClBr$) 297.9555, measured 297.9551, 1.34 ppm error
IR (neat) vmax/cm^{-1} 2955, 2923, 2852, 1581, 1467, 1372, 1217, 1084, 1073, 1004, 811

Competition Experiment Results

Run	**2g** / % (pDC)	**2g** / % (DC)
1	30	25
2	34	30
3	35	27
Average	33	27.3
Standard Deviation	2.65	2.52

1-(tert-Butyl)-3-(1-fluoroethyl)benzene **2h**

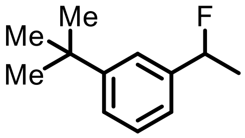

1-(tert-Butyl)-3-(1-fluoroethyl)benzene was prepared from methyl 1-(tert-butyl)-3-ethylbenzene via general procedure 2 and purified by column chromatography (1% ethyl acetate in pentane) to afford the title product as an off-white oil.

^{1}H NMR (400 MHz, $CDCl_3$) δ 7.39 – 7.29 (m, 3H), 7.21 – 7.13 (m, 1H), 5.63 (dq, *J* = 47.7, 6.4 Hz, 1H), 1.66 (dd, *J* = 23.9, 6.4 Hz, 3H), 1.34 (s, 9H).
^{19}F NMR (376 MHz, $CDCl_3$) δ -165.78 (dq, *J* = 47.8, 23.9 Hz).
^{13}C NMR (151 MHz, $CDCl_3$) δ 151.6, 141.2 (d, *J* = 19.2 Hz), 128.3, 125.4 (d, *J* = 1.9 Hz), 122.5 (d, *J* = 6.4 Hz), 122.4 (d, *J* = 6.7 Hz), 91.5 (d, *J* = 167.0 Hz), 34.9, 31.5, 23.1 (d, *J* = 25.4 Hz).
Data are in agreement with the literature.[6]

Competition Experiment Results

Run	**2h** / % (pDC)	**2h** / % (DC)
1	63	50
2	67	50
3	73	61
Average	67.7	53.7
Standard Deviation	5.03	6.35

<u>A note on product isolation and handling:</u> Due to common stability issues regarding isolation and storage of secondary benzyl fluorides, upon completion of electrolysis the crude reaction mixture was immediately transferred to a HDPE container and NMR yields were determined from the crude reaction mixture using NMR tubes with PTFE liners to avoid decomposition of the products.

2h could be isolated via column chromatography and was subsequently stored in a HDPE vial.

1-Fluoro-2,3-dihydro-1H-indene **2i**

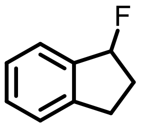

1-Fluoro-2,3-dihydro-1H-indene was prepared from 2,3,-dihydro-1H-indene via general procedure 2. The final product could not be isolated via column chromatography on silica gel. ^{19}F NMR shifts for determining NMR yields were therefore assigned based on previous literature reports identifying the benzyl fluoride shift in crude reaction mixtures.[7] A representative crude NMR is presented in section 9.

[Crude] **^{19}F {^{1}H} NMR** (377 MHz) -160.0 (s)

Competition Experiment Results

Run	**2i** / % (pDC)	**2i** / % (DC)
1	62	41
2	54	43
3	62	44
Average	59.3	42. 7
Standard Deviation	4.62	1.53

7. Electrode Information

Electrode preparation

Platinum electrodes were prepared via a procedure previously reported by our group.[8] 15 cm of 0.5 mm thickness platinum wire (purchased from Advent Research Materials – see general considerations) was coiled around PTFE tubing to create a surface area of approximately 1 cm^2. The other end was inserted into a wider PTFE tubing and soldered to a copper wire. The wire was passed through a B24 Suba-Seal.

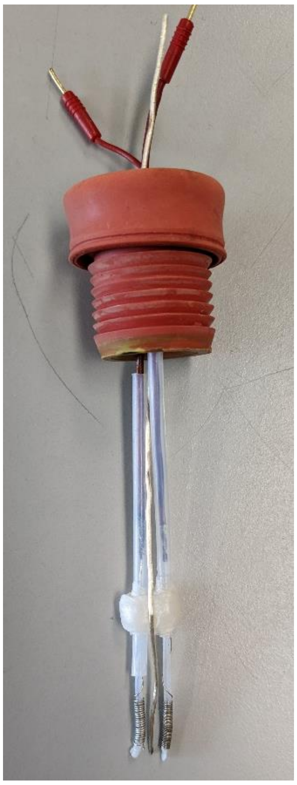

Figure S4. Platinum | Platinum | Ag wire electrolysis electrodes

Electrode cleaning

After electrolysis the electrodes were allowed to submerge in sat. aq. $NaHCO_3$ for 10 minutes before rinsing with H_2O before wiping and rinsing with acetone. In the case where electrodes were not cleaned sufficiently after this initial wash, electrodes were submerging in a solution of 0.1 M $TBAPF_6$ in MeCN and applying a fixed cell potential of 2.5 V across the electrodes for 30 seconds alternating each electrode as the working and counter electrode. Alternatively, the platinum wire could be uncoiled and wiped with an acetone-soaked paper towel.

Cell setup

Electrolysis was conducted in a standard Young's tube. These could be used with Et_3N•3HF repeatedly without any issue.

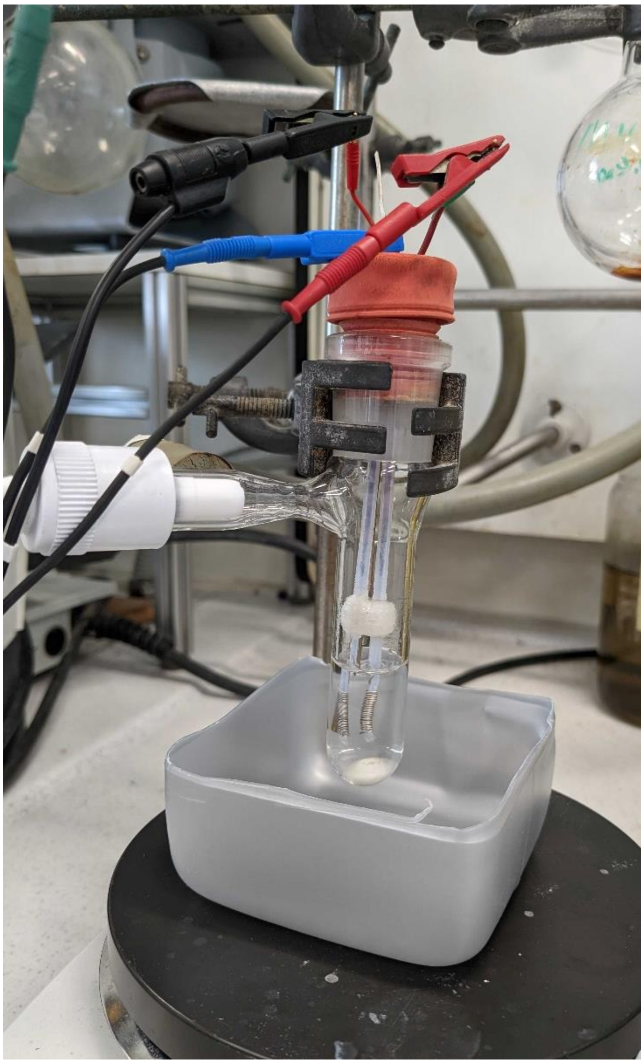

Figure S5. Young's flask with stirrer bar and electrodes connected to potentiostat (working electrode, counter electrode and reference electrode).

8. Mechanistic Rationale for Pulsed Electrolysis

Role of pulsing in reducing overoxidation of benzyl fluoride product

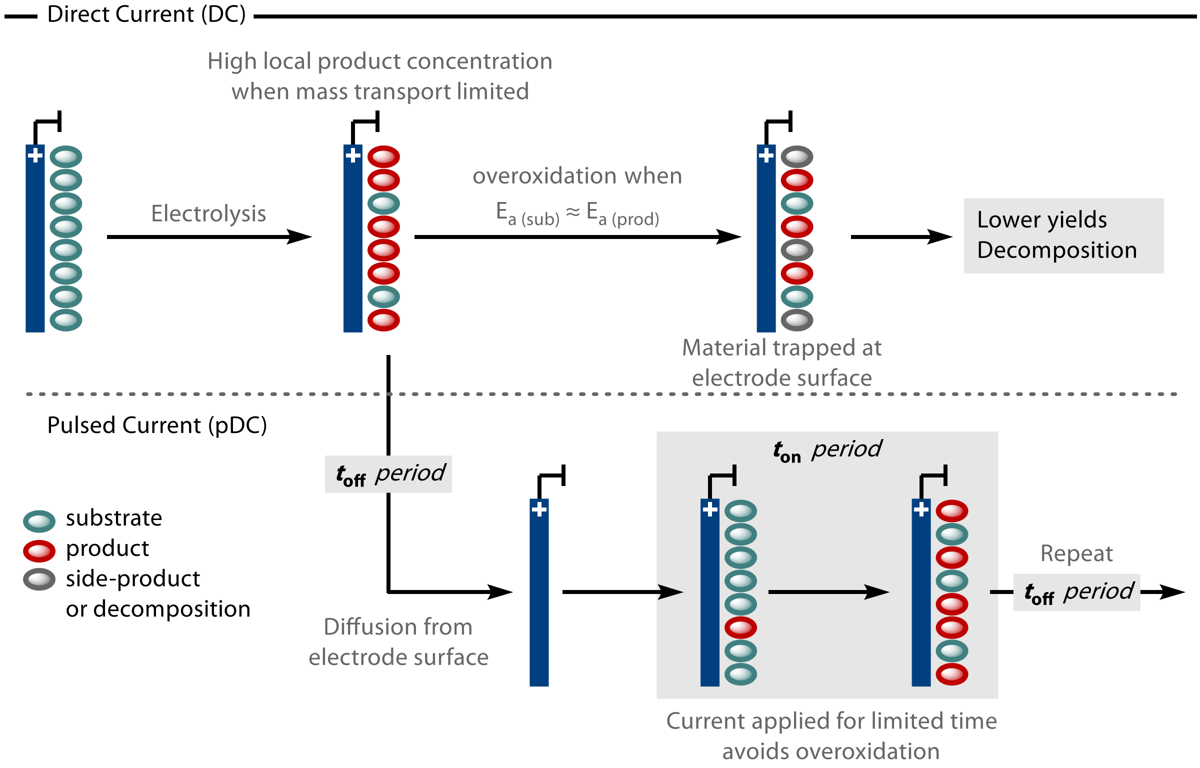

Figure S6. Cartoon schematic exploring effect of using a pulsed electrolysis in avoiding overoxidation of product.

During DC electrolysis substrate at the anodic surface undergoes the proposed ET/PT/ET process to generate the benzylic cation that is subsequently attacked by fluoride to form product, but limitations in mass transport (suspected due to formation of an insulating layer in the electrical double layer) arrests the removal of material from the vicinity of the electrode surface resulting in overoxidation and reduced yields and mass balance (DC regime). However, by changing regime to a pulsed electrolysis waveform and including a t_{off} sequence allows diffusion of the trapped species (interruption of the electrostatic interactions forming the insulating layer) away from the electrode surface (as observed by the decreasing potential observed during t_{off} and during OCP measurements). Limiting the duration of the applied current to the t_{on} duration by including the t_{off} period avoids the overoxidation of product, resulting in increased yields and mass balances.

9. Spectra of novel compounds

Methyl (4’-methyl-[1,1’-biphenyl]-4-yl) carbonate ***1b***

¹H NMR (400 MHz, $CDCl_3$)

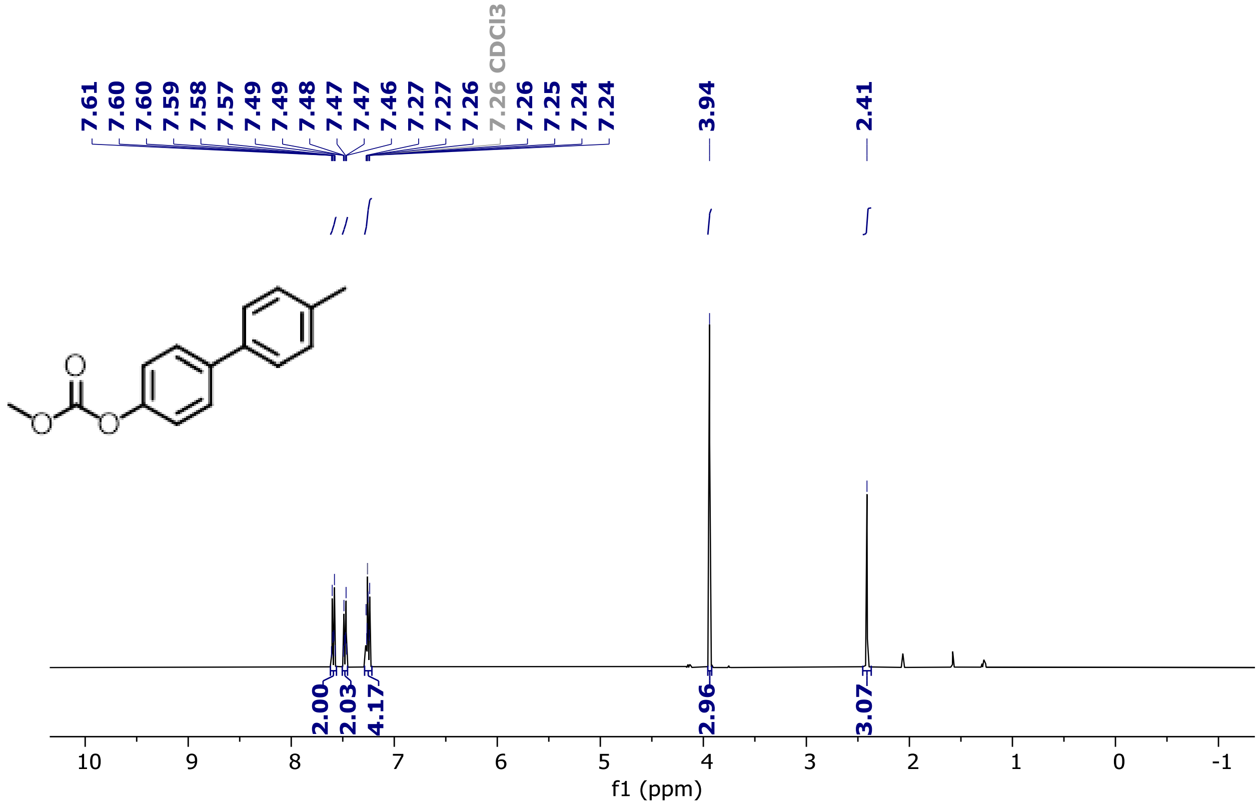

¹³C NMR (101 MHz, $CDCl_3$)

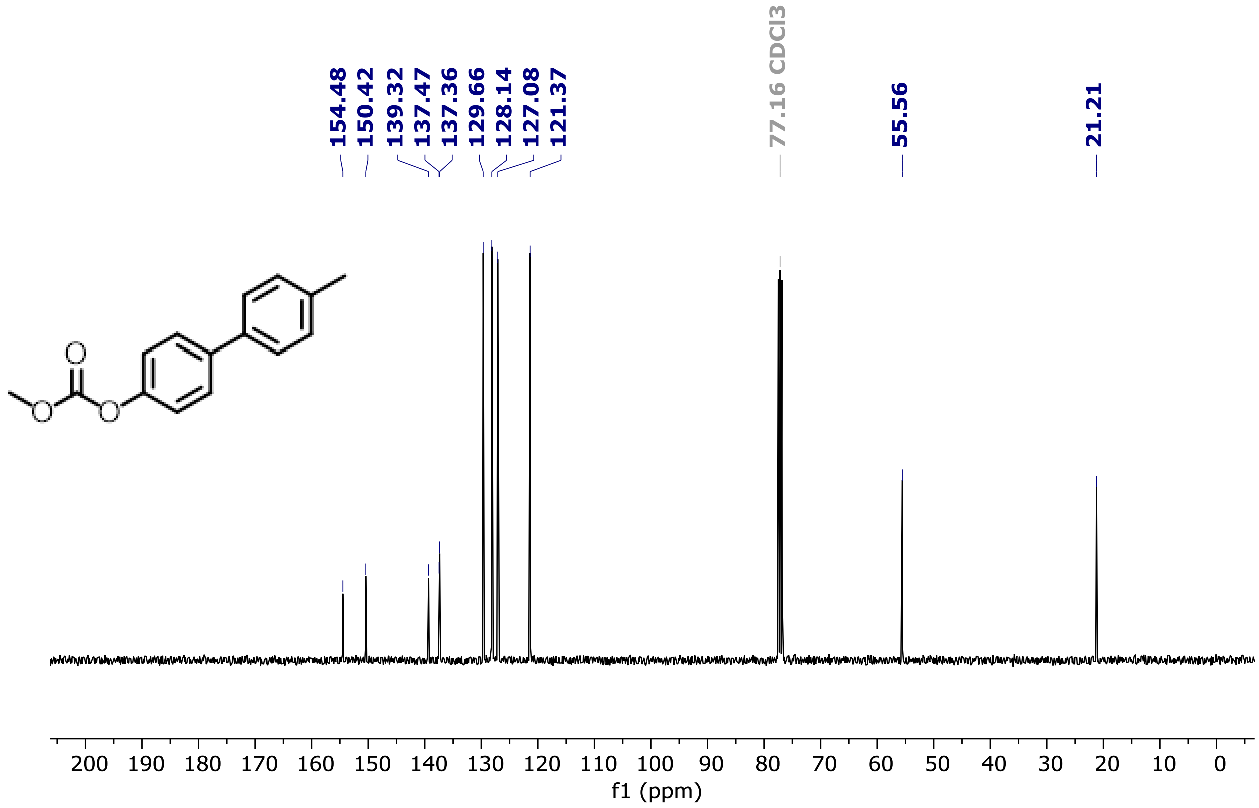

3'-Bromo-5'-fluoro-2-methyl-1,1'-biphenyl ***1f***

^{1}H NMR (400 MHz, $CDCl_3$)

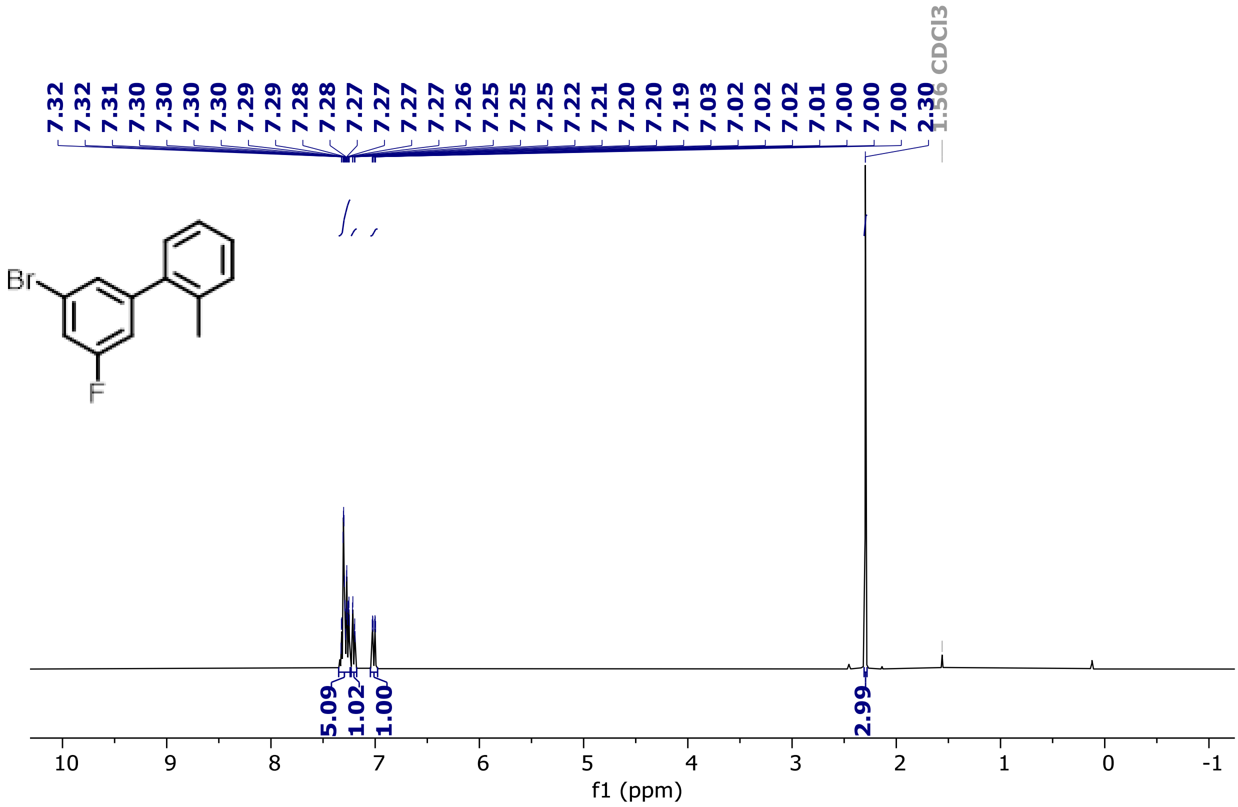

^{19}F NMR (377 MHz, $CDCl_3$)

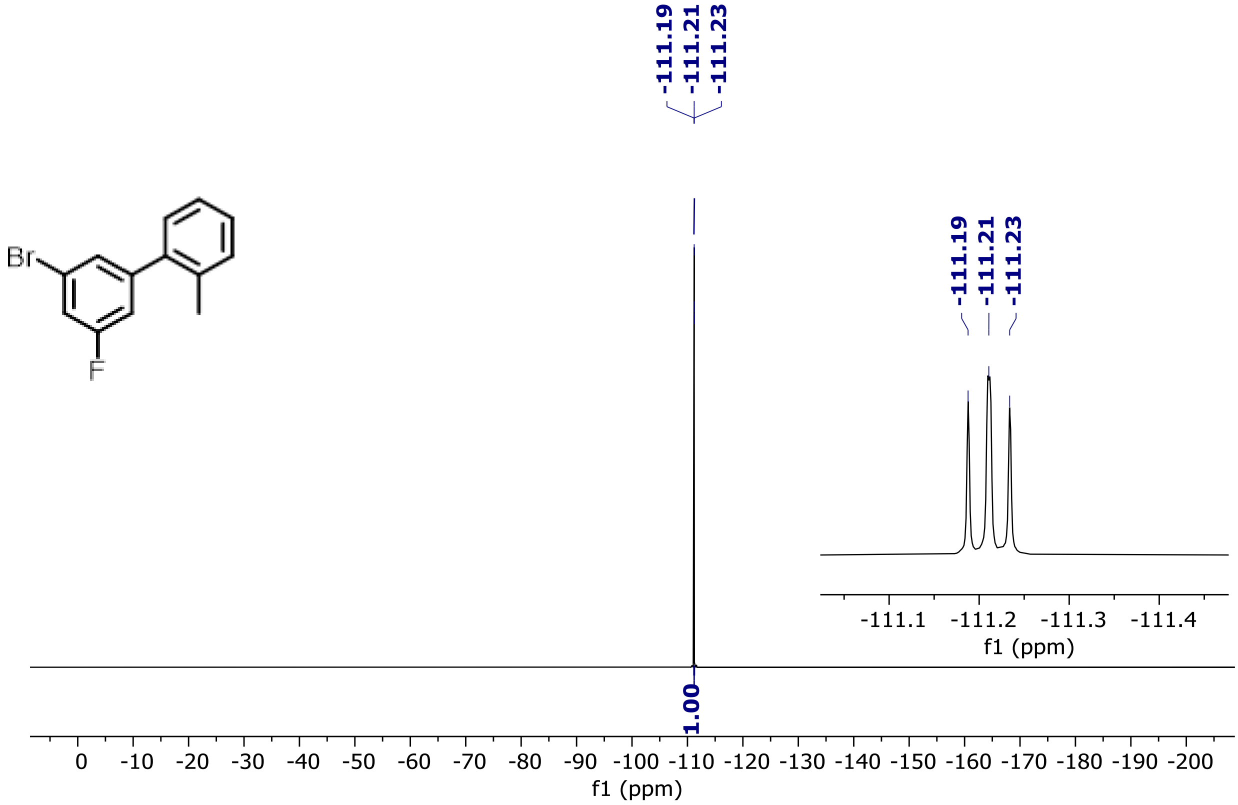

^{13}C NMR (101 MHz, $CDCl_3$)

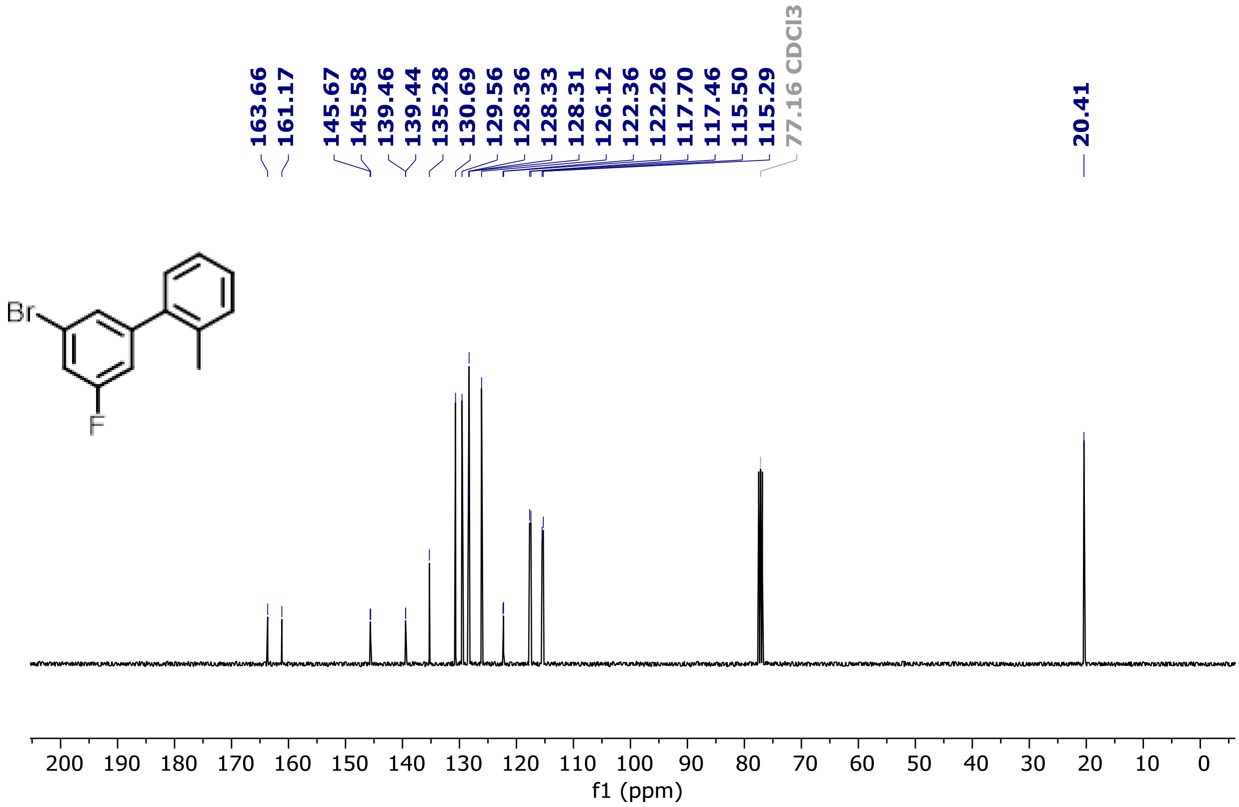

4-Bromo-2-chloro-4'-methyl-1,1'-biphenyl ***1g***

^{1}H NMR (400 MHz, $CDCl_3$)

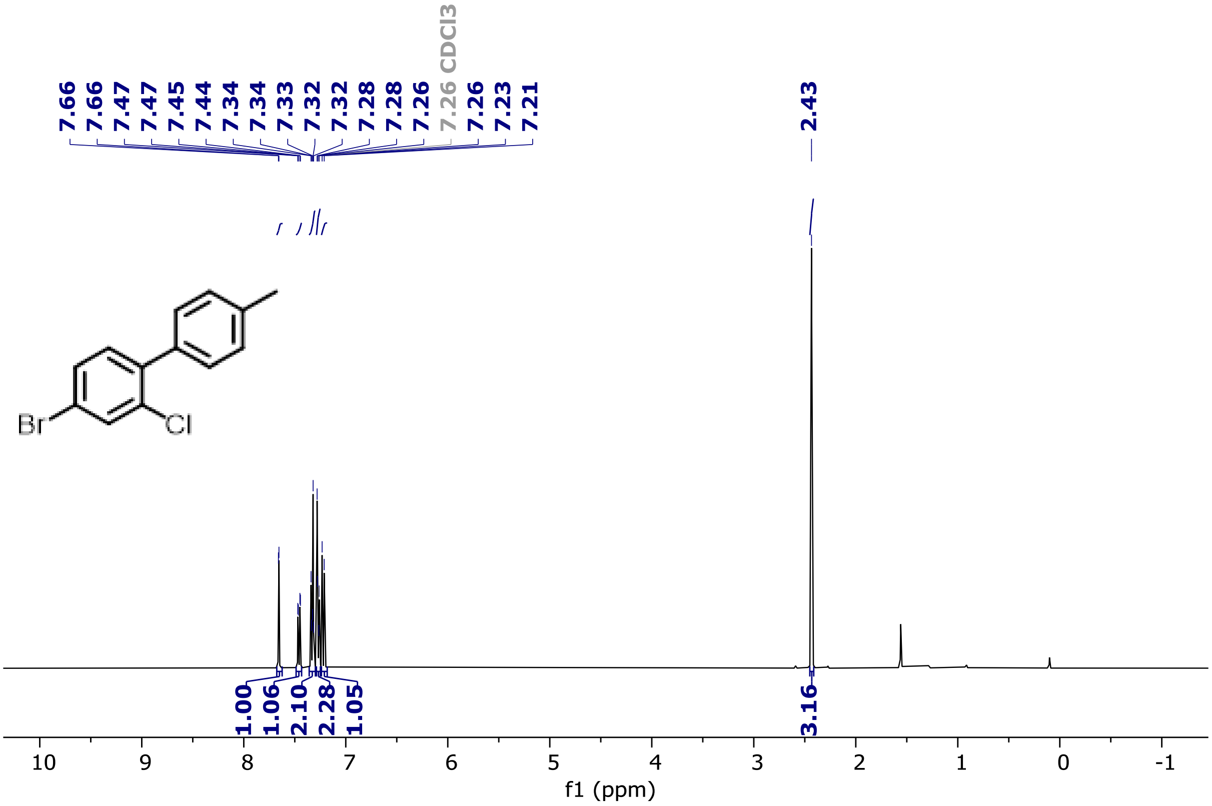

^{13}C NMR (101 MHz, $CDCl_3$)

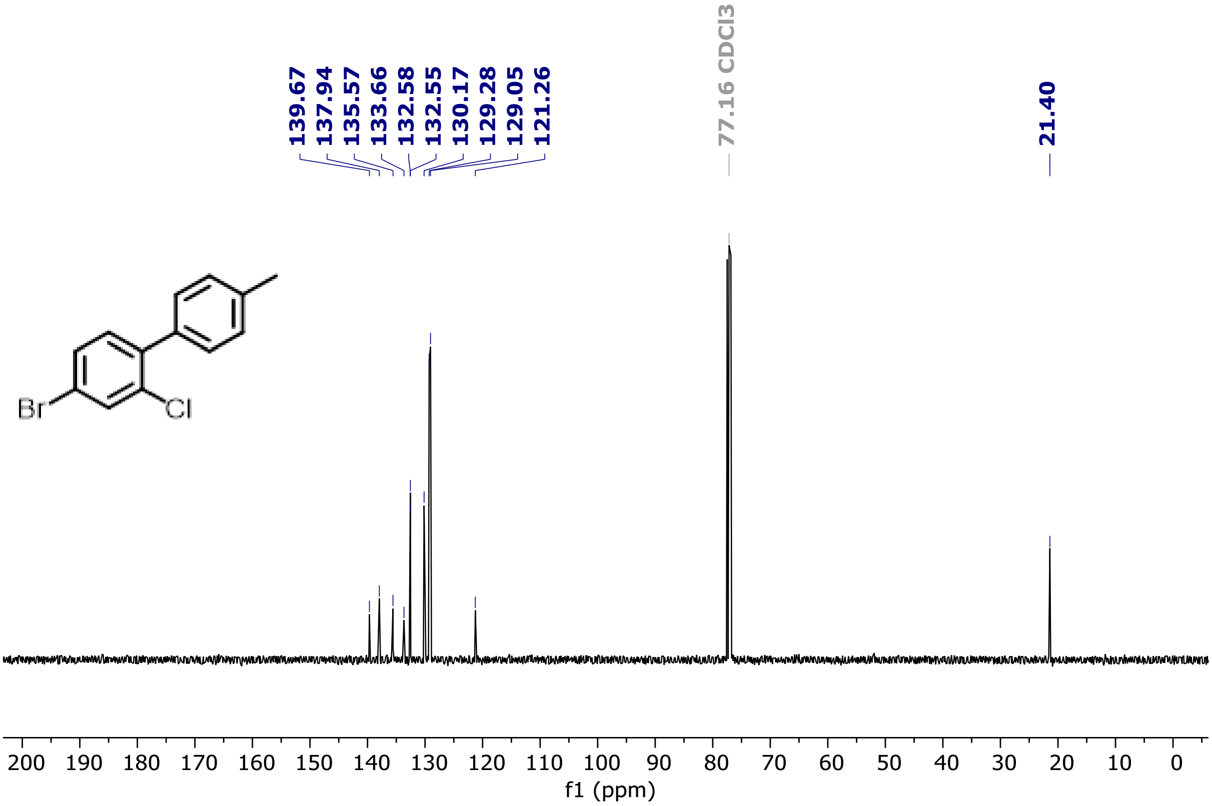

4-Fluoro-4’(fluoromethyl)-1,1’-biphenyl **2a**

^{1}**H NMR** (400 MHz, $CDCl_3$)

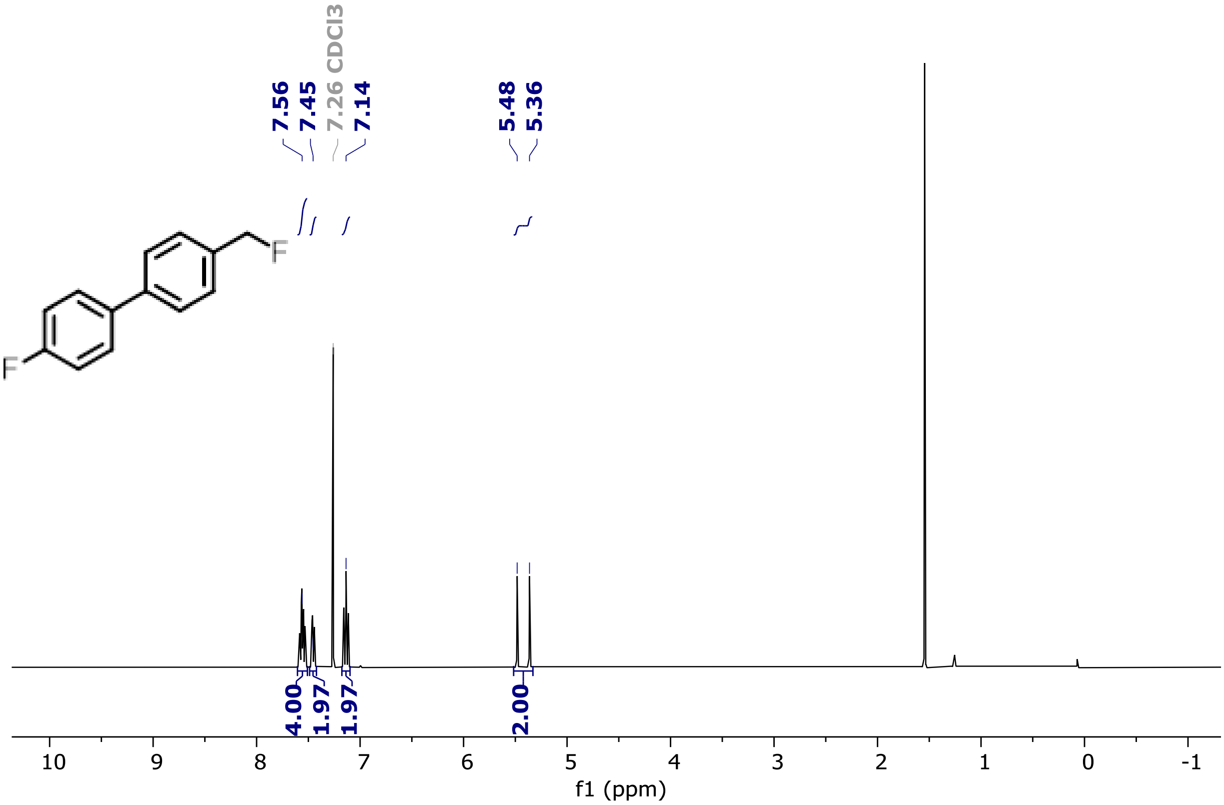

^{19}**F NMR** (377 MHz, $CDCl_3$)

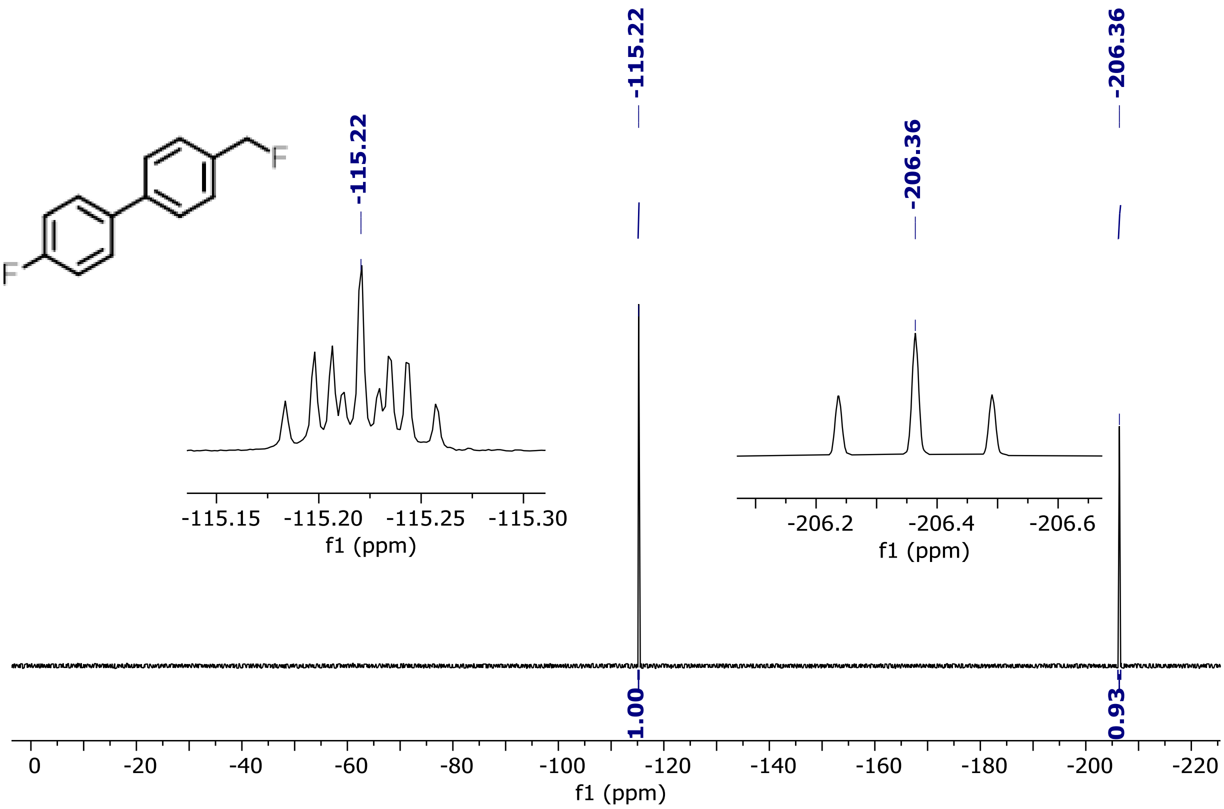

^{13}C NMR (101 MHz, $CDCl_3$)

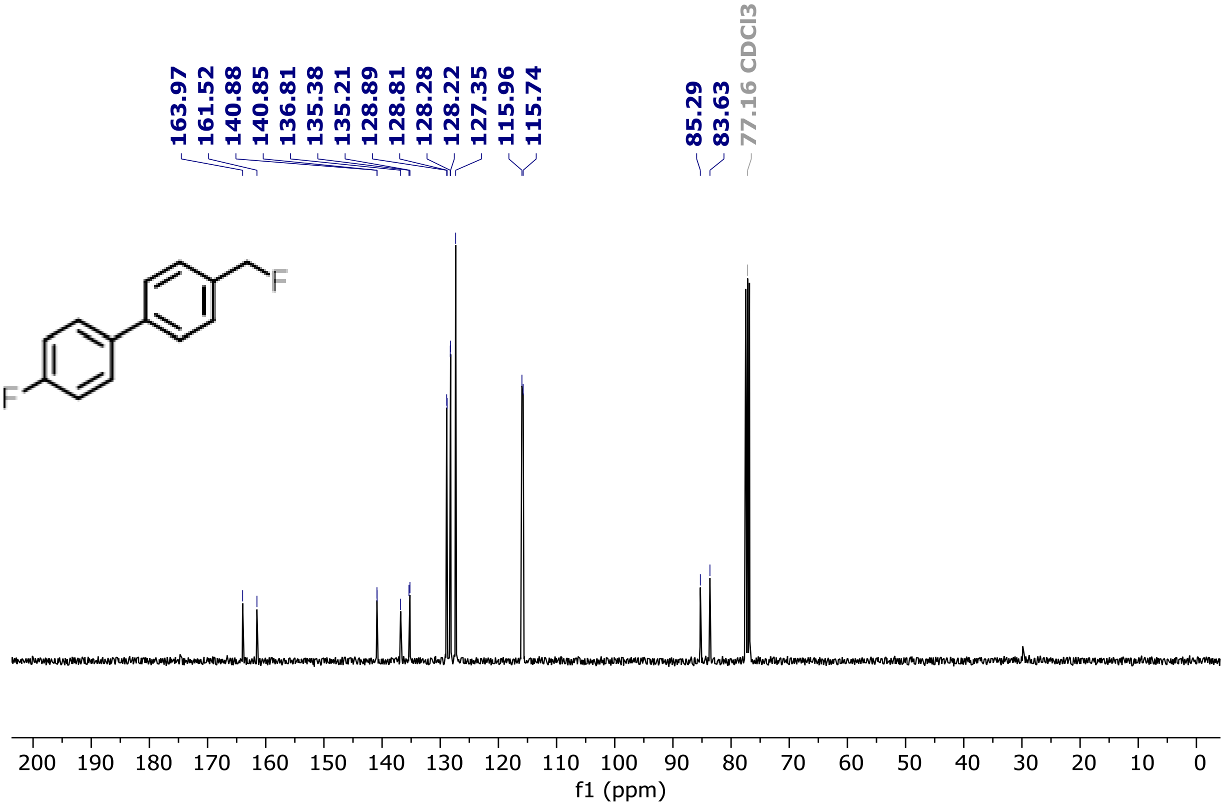

4'-(Fluoromethyl)-[1,1'-biphenyl]-4-yl methyl carbonate **2b**

^{1}H NMR (400 MHz, $CDCl_3$)

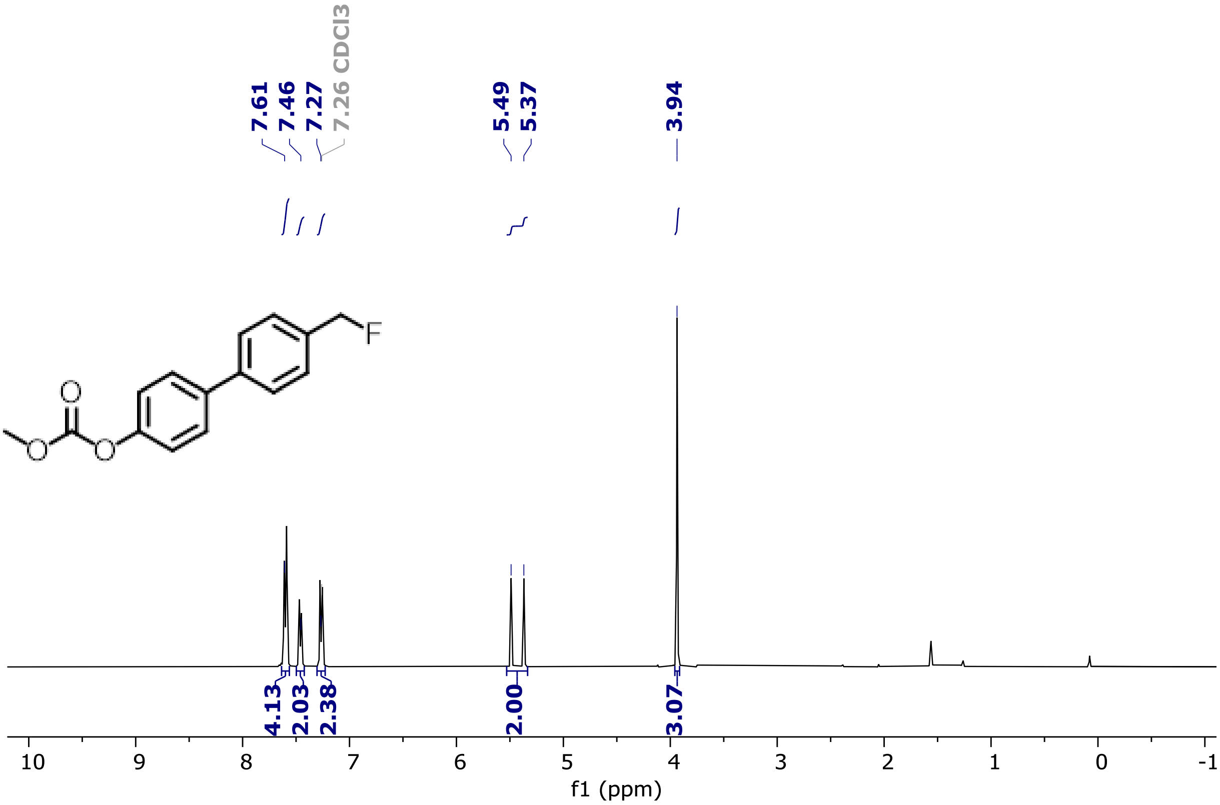

^{19}F NMR (376 MHz, $CDCl_3$)

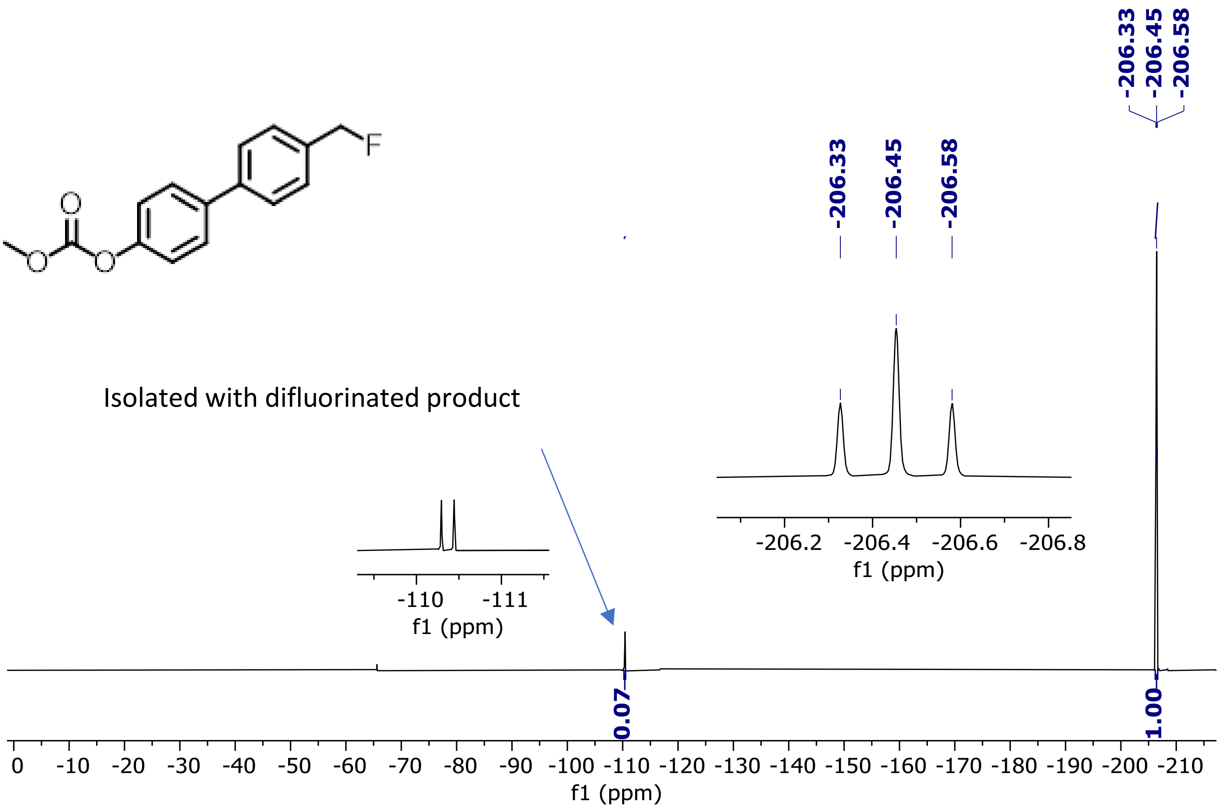

^{13}C NMR (151 MHz, $CDCl_3$)

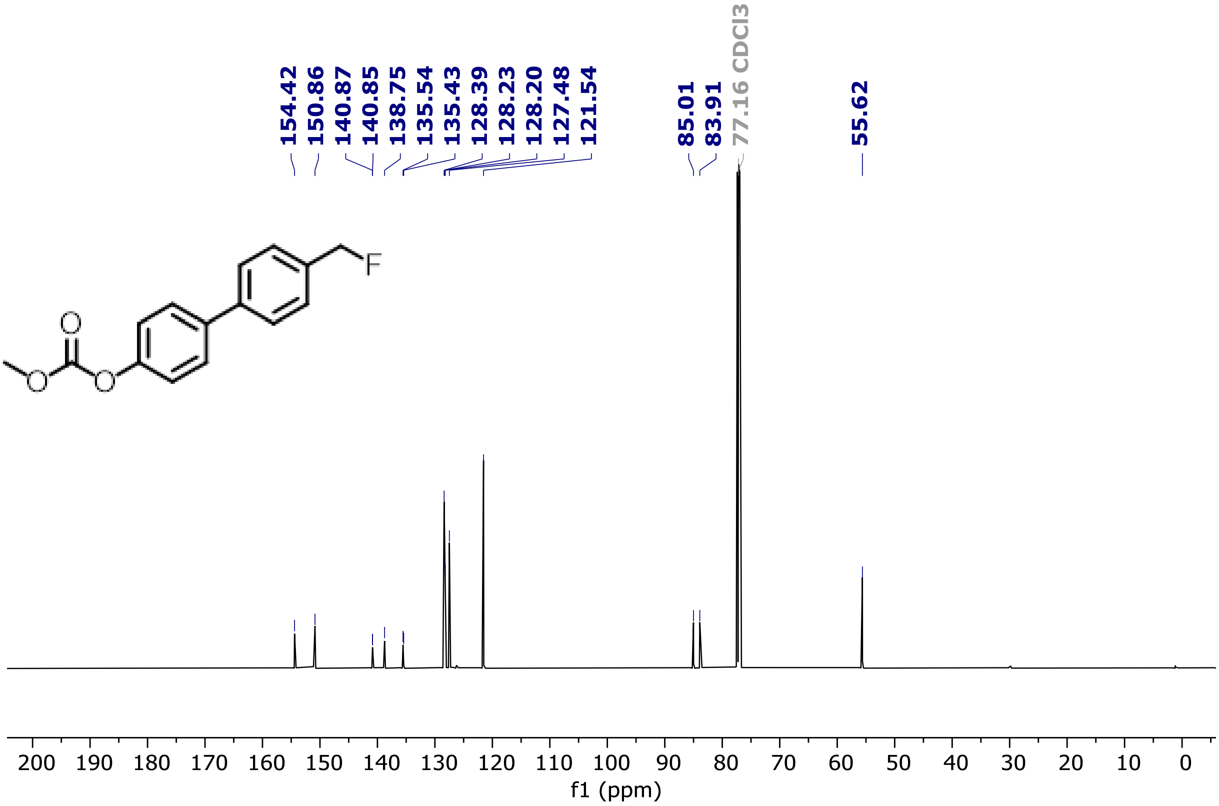

4-Bromo-4'-(fluoromethyl)-1,1'-biphenyl **2c**

^{1}H NMR (400 MHz, $CDCl_3$)

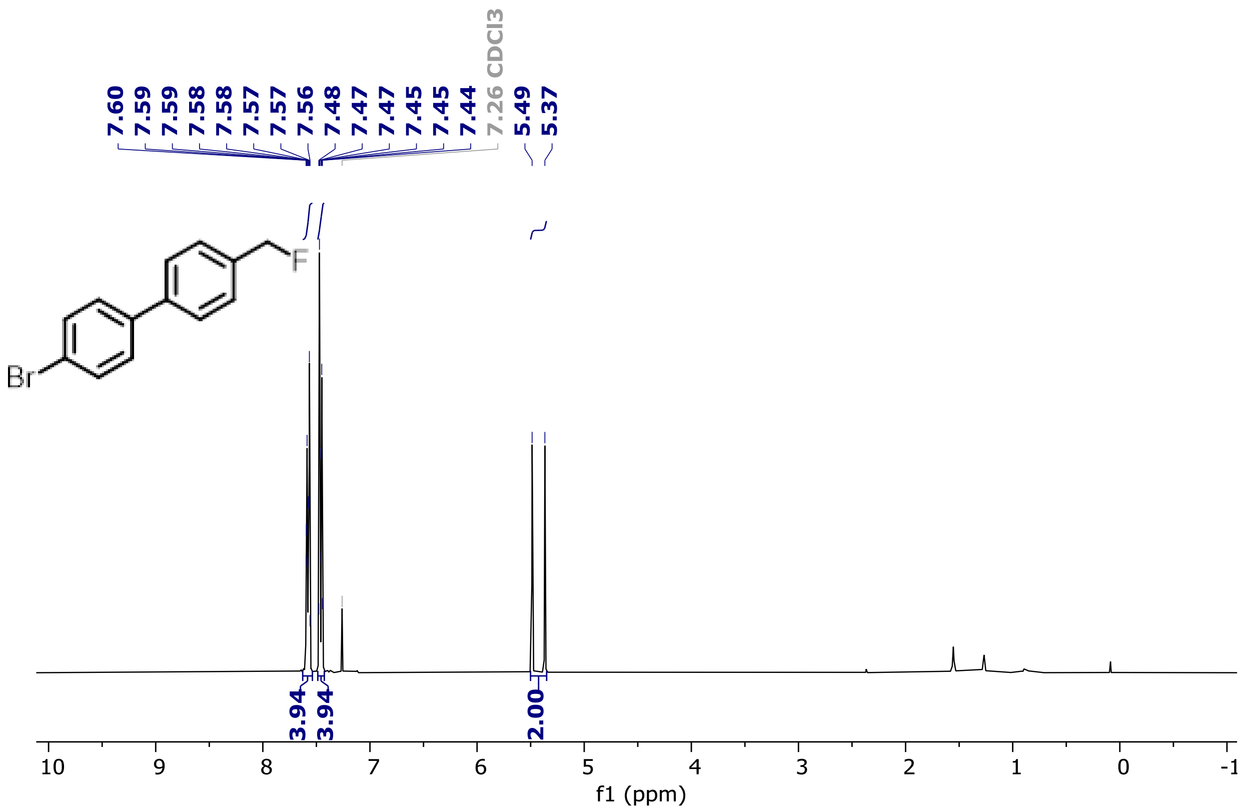

^{19}F NMR (377 MHz, $CDCl_3$)

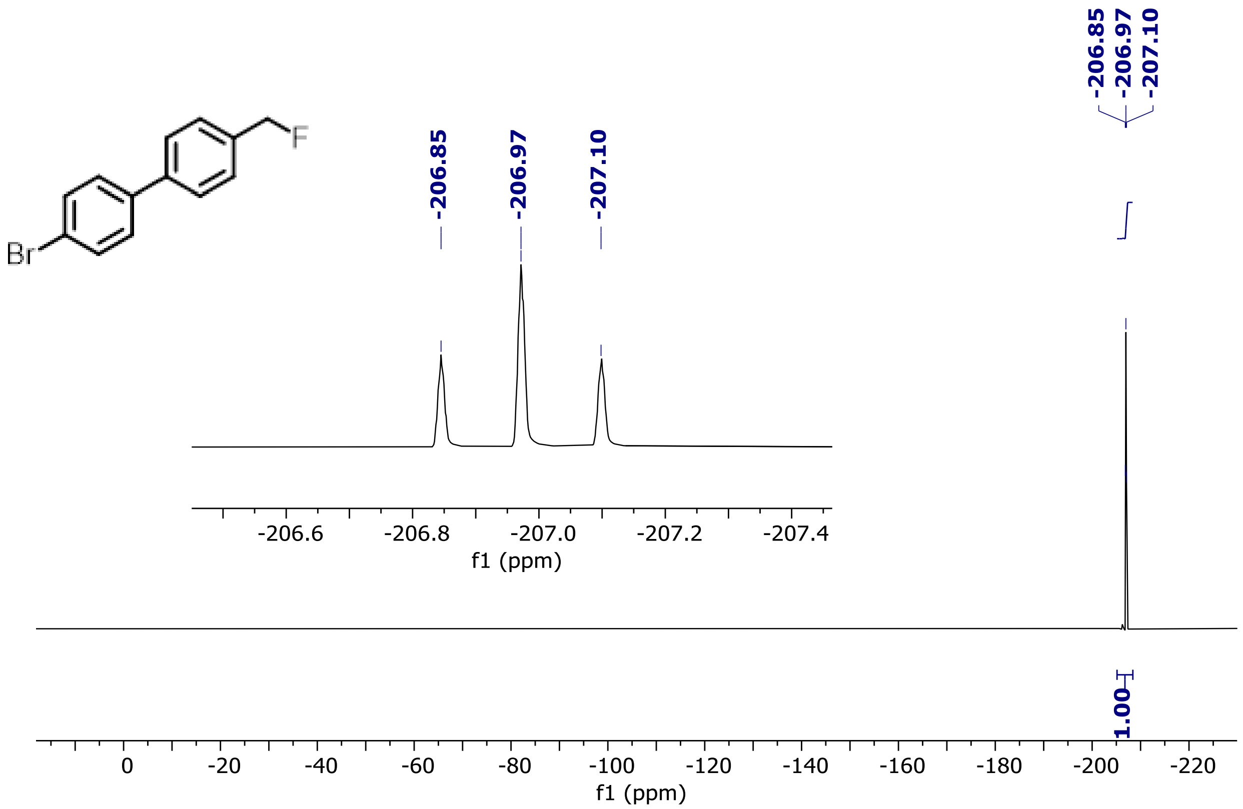

^{13}C NMR (101 MHz, $CDCl_3$)

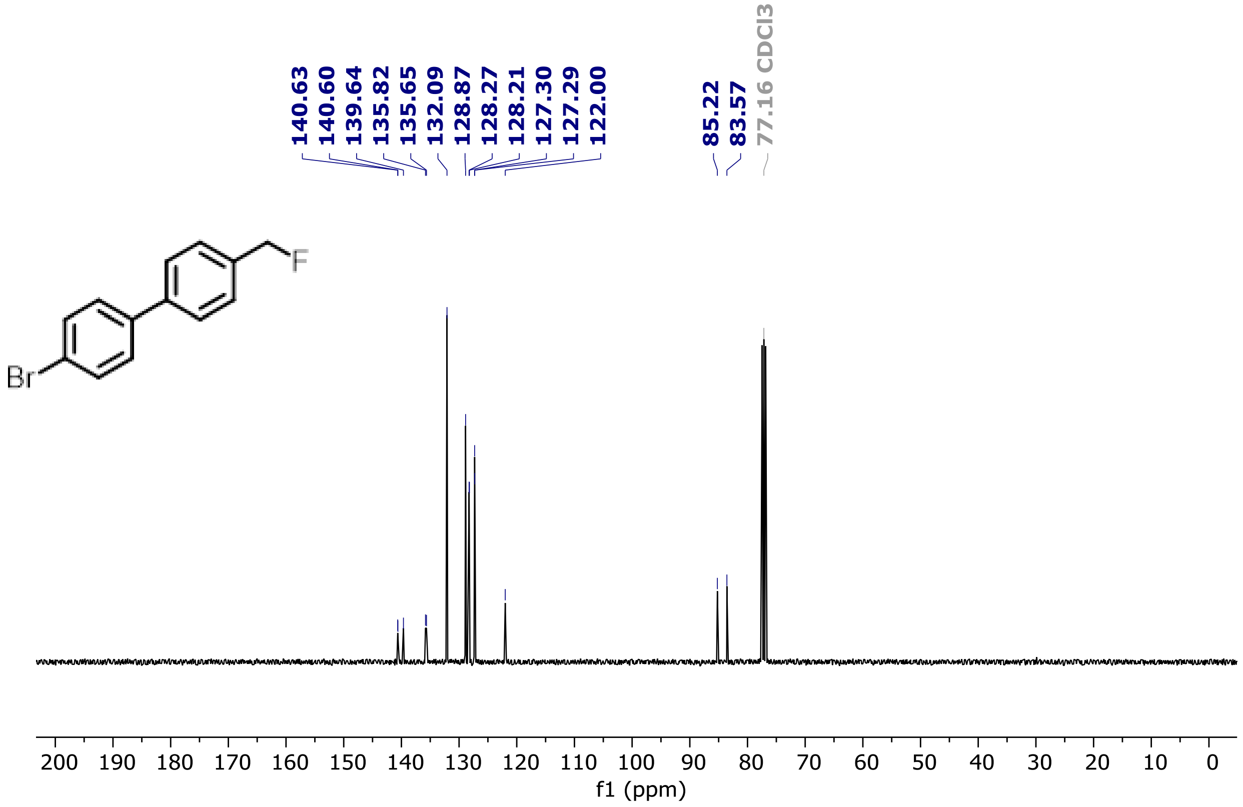

4-(Fluoromethyl)-4'-(trifluoromethyl)-1,1'-biphenyl **2d**

^{1}H NMR (400 MHz, $CDCl_3$)

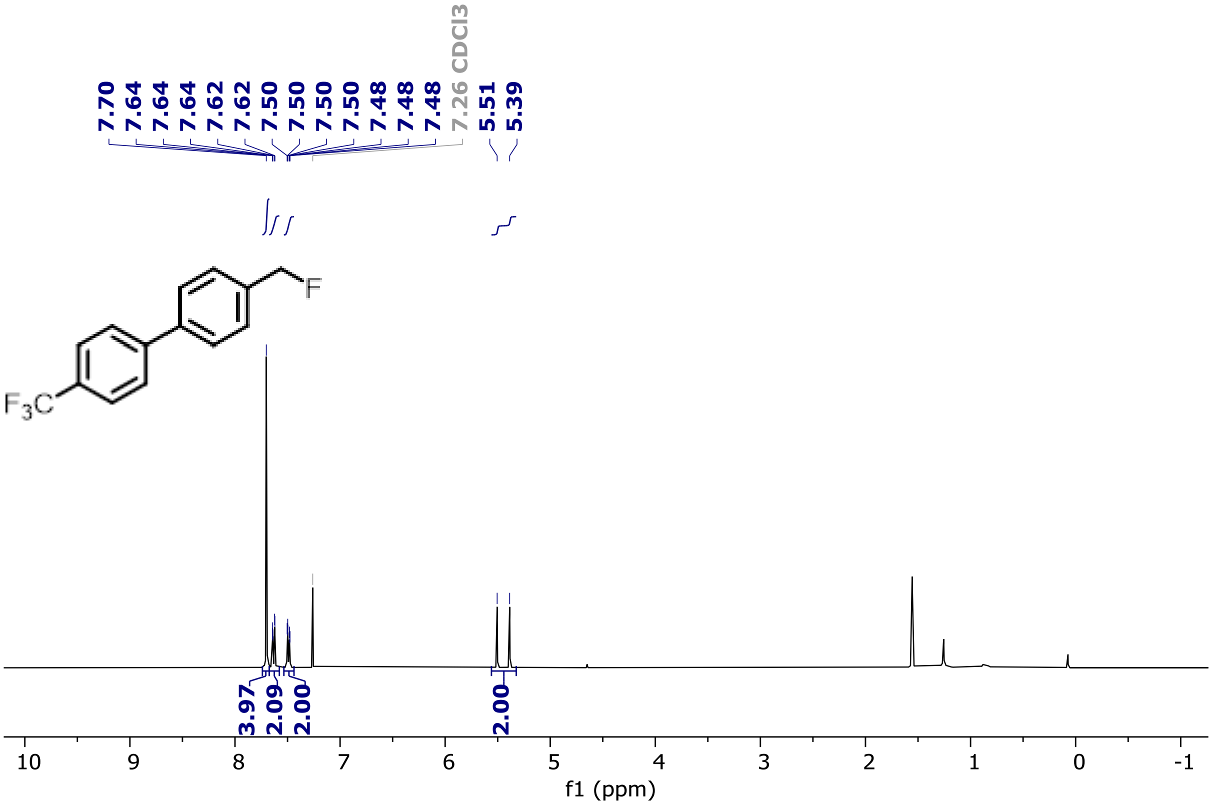

^{19}F NMR (377 MHz, $CDCl_3$)

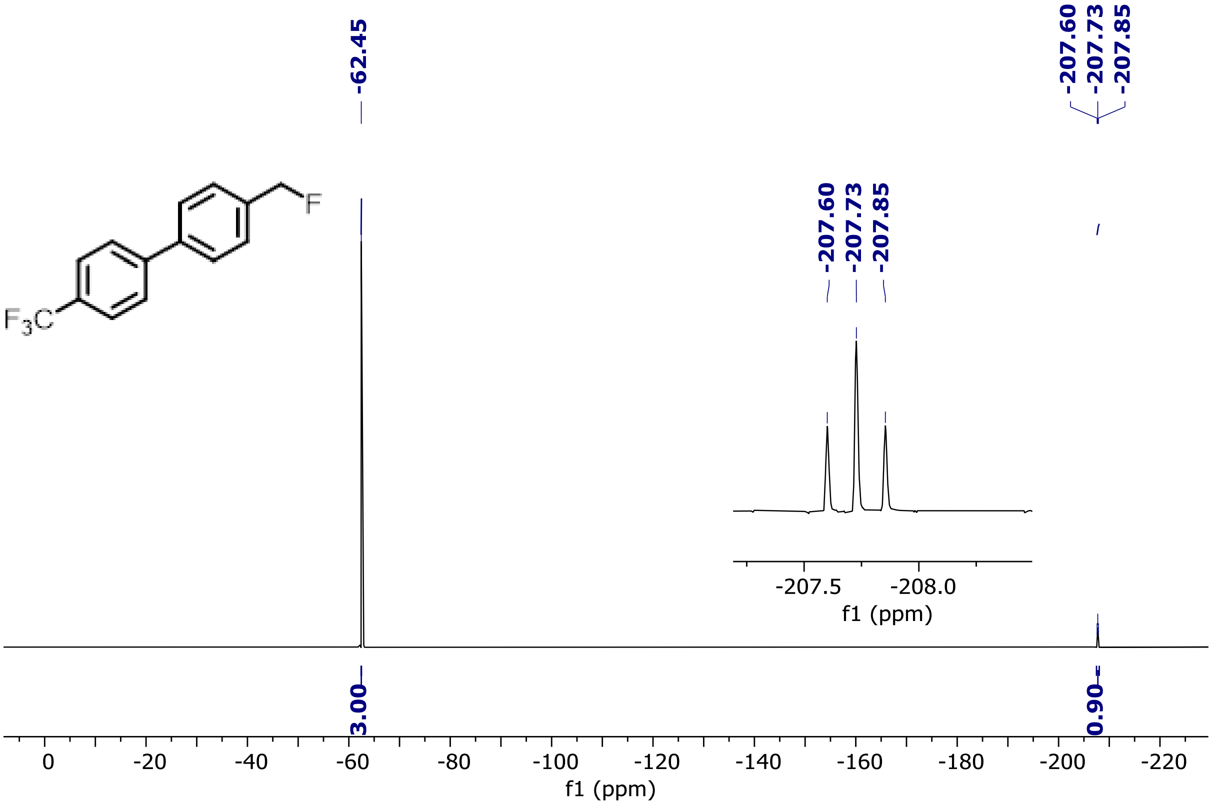

^{13}C NMR (151 MHz, $CDCl_3$)

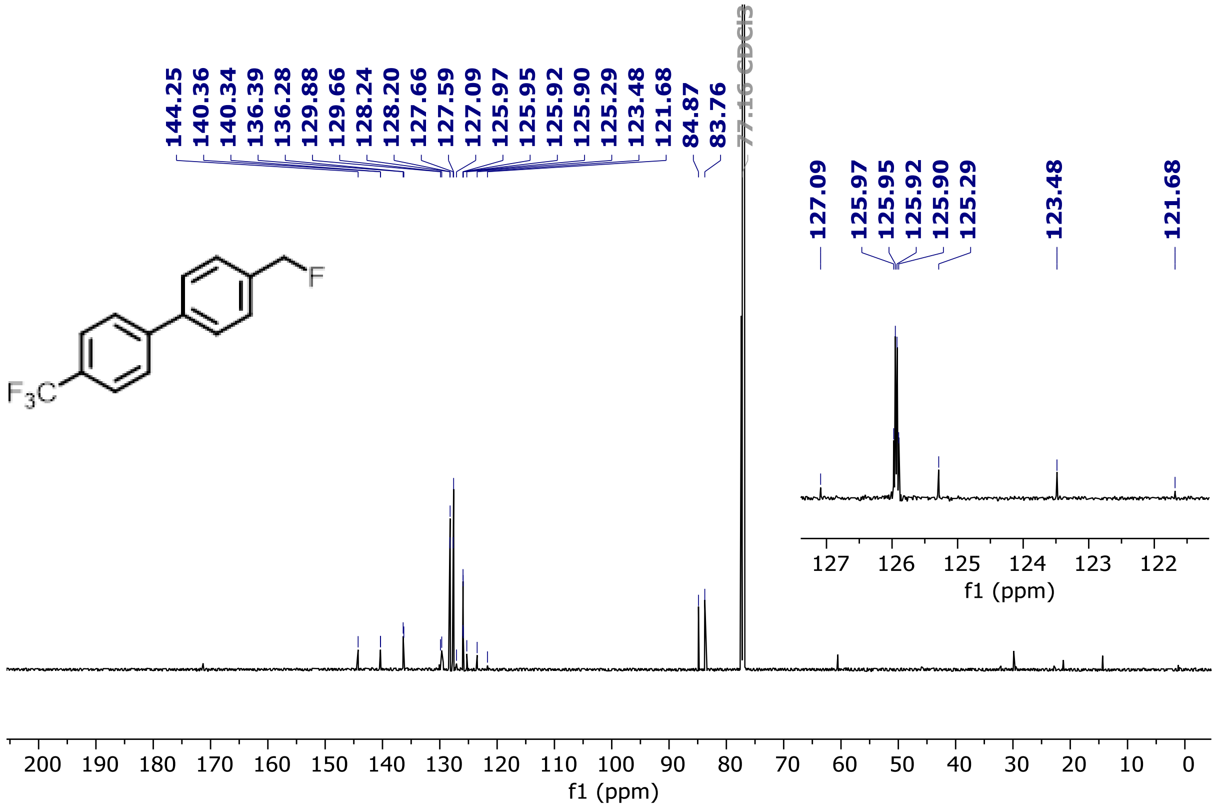

4-Chloro-4'-(fluoromethyl)-1,1'-biphenyl **2e**

^{1}H NMR (400 MHz, $CDCl_3$)

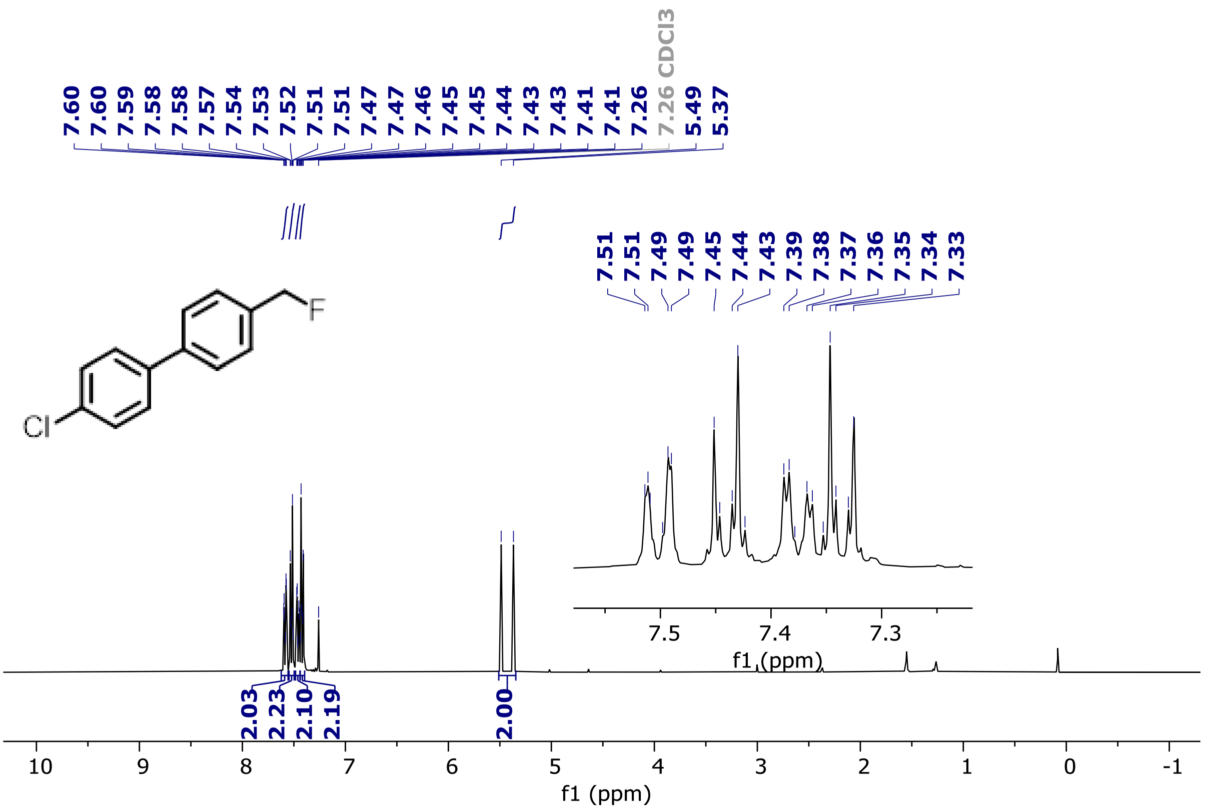

^{19}F NMR (377 MHz, $CDCl_3$)

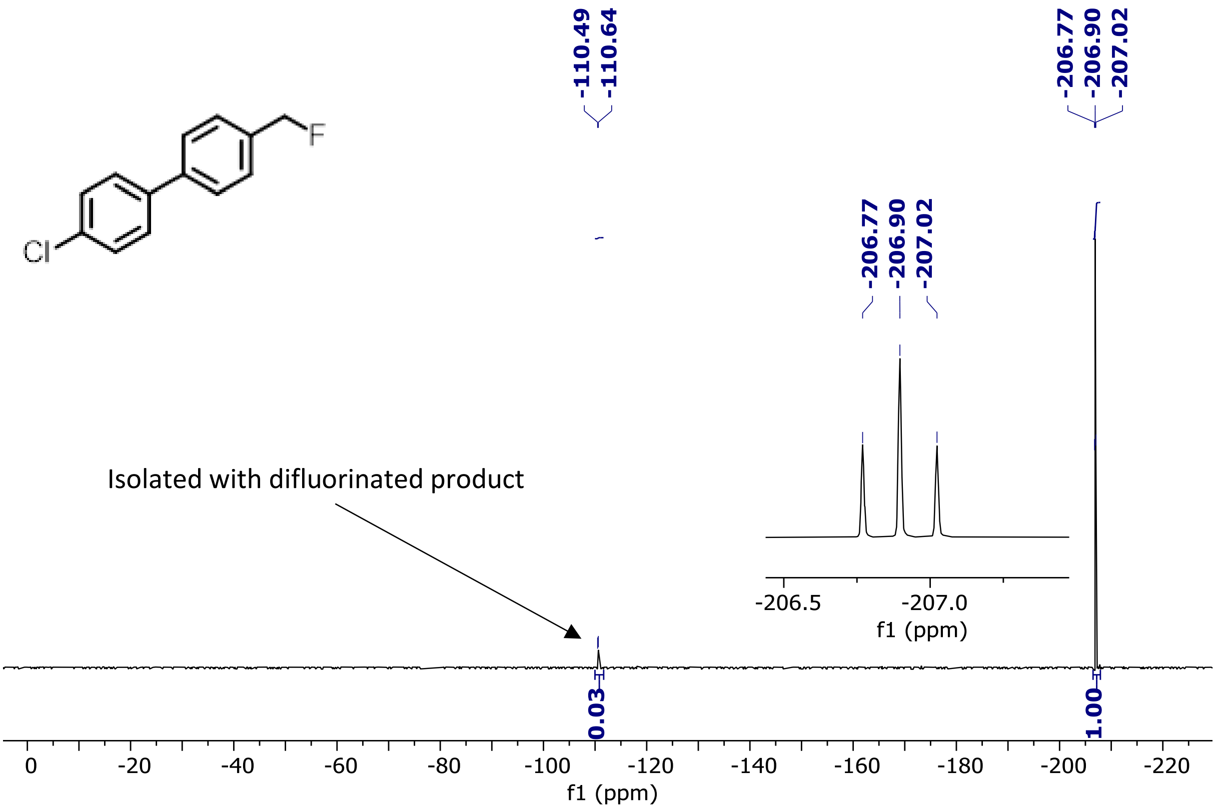

^{13}C NMR (101 MHz, $CDCl_3$)

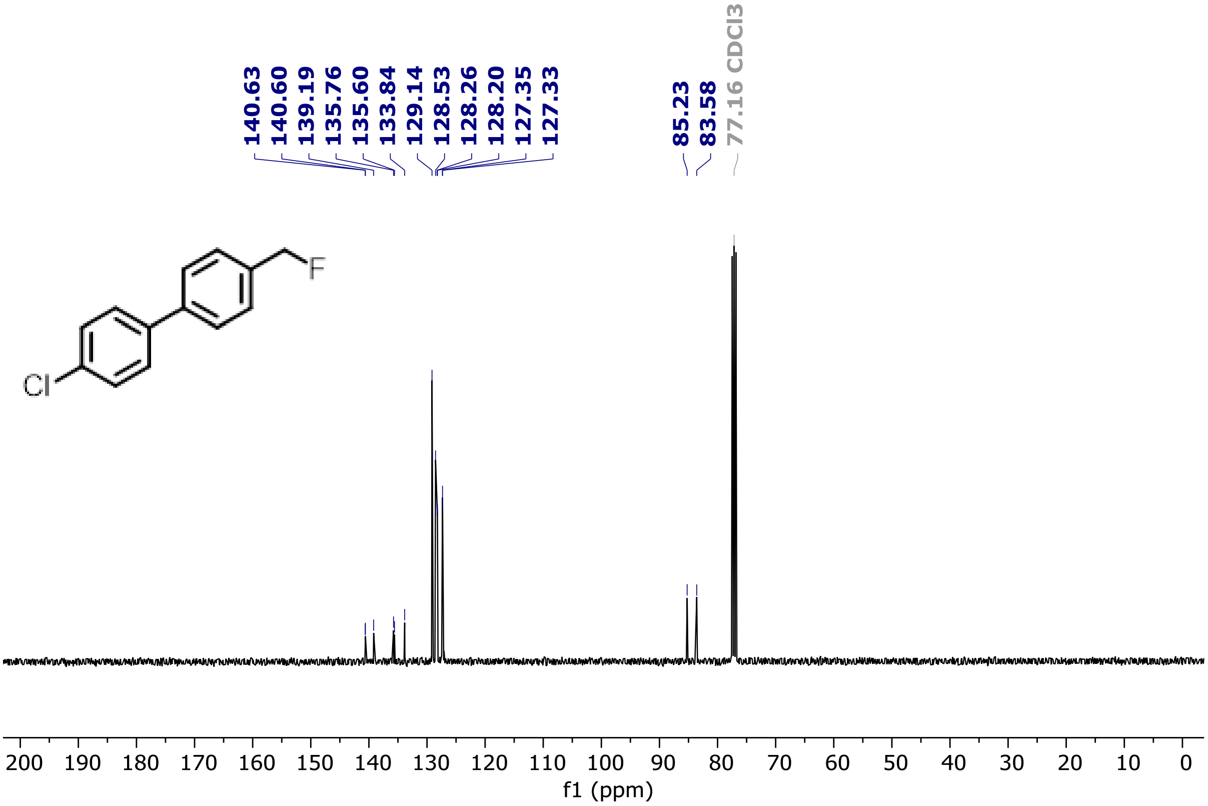

3'-Bromo-5'-fluoro-2-(fluoromethyl)-1,1'-biphenyl **2f**

^{1}H NMR (400 MHz, $CDCl_3$)

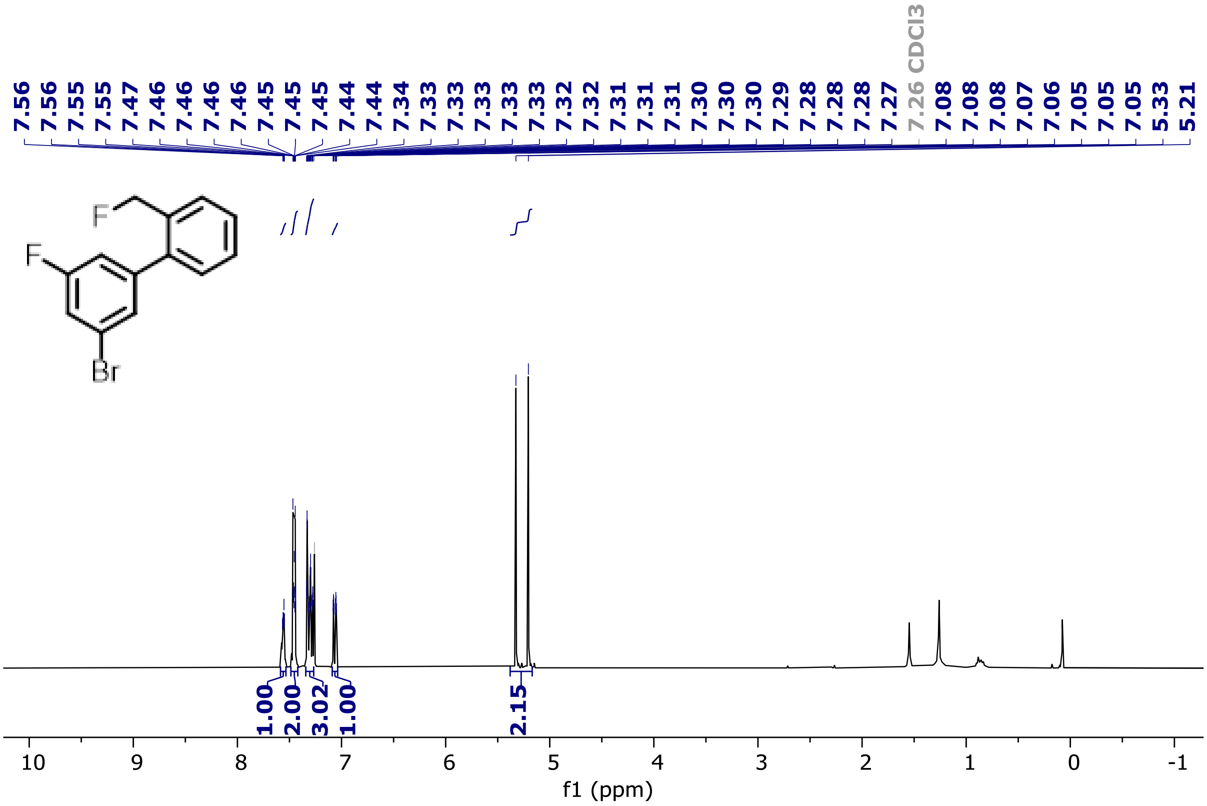

^{19}F NMR (377 MHz, $CDCl_3$)

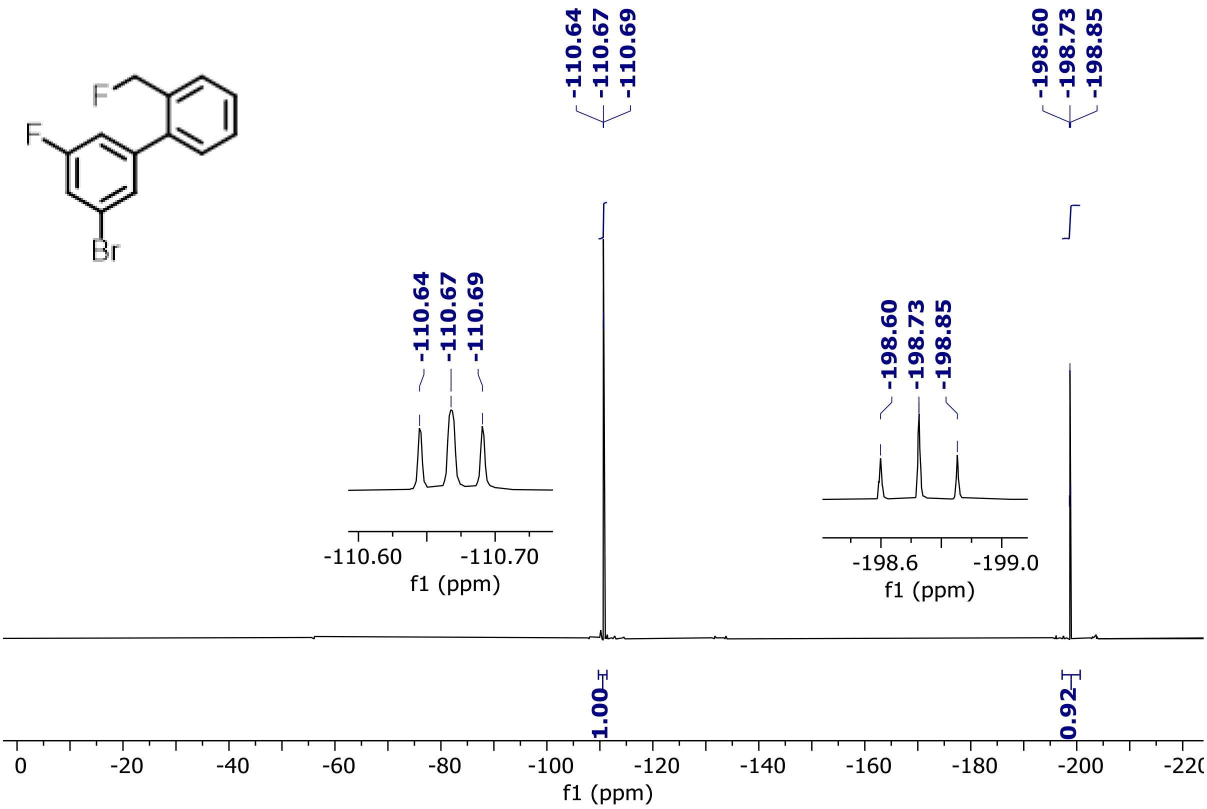

^{13}C NMR (101 MHz, $CDCl_3$)

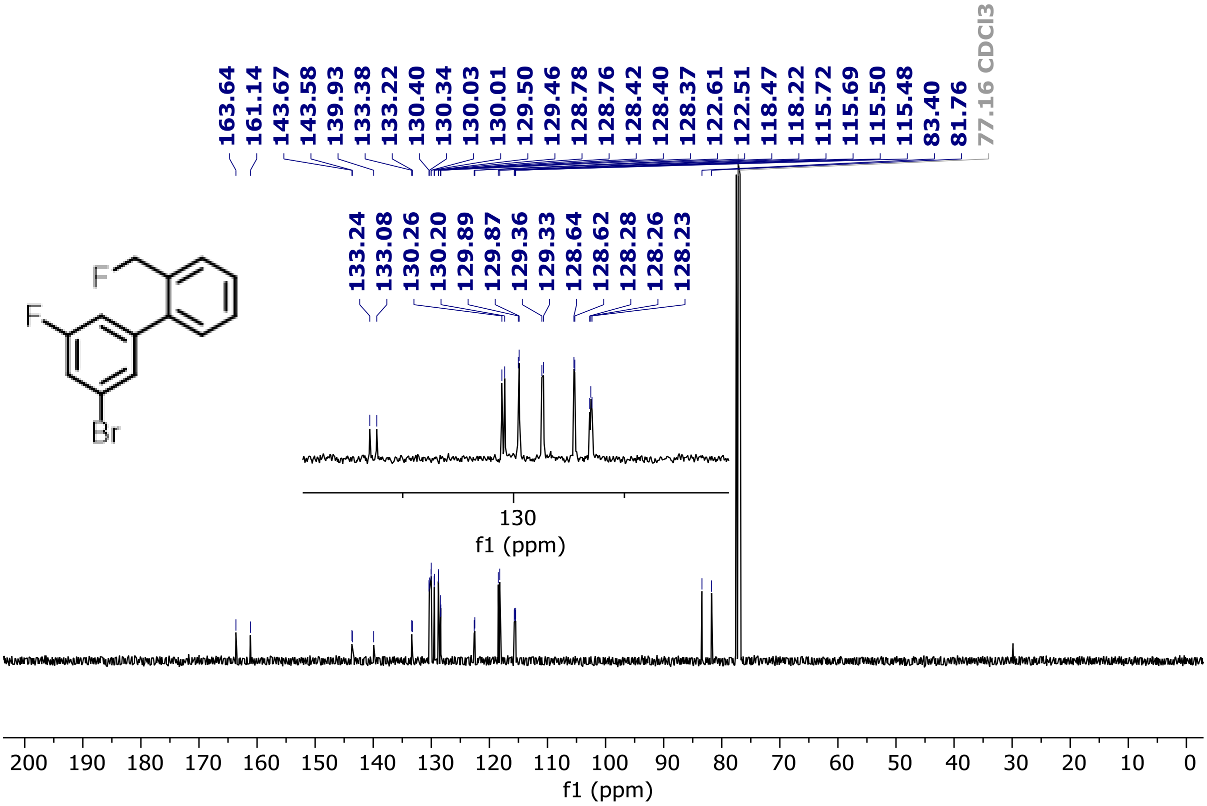

4-Bromo-2-chloro-4'-(fluoromethyl)-1,1'-biphenyl **2g**

^{1}H NMR (400 MHz, $CDCl_3$)

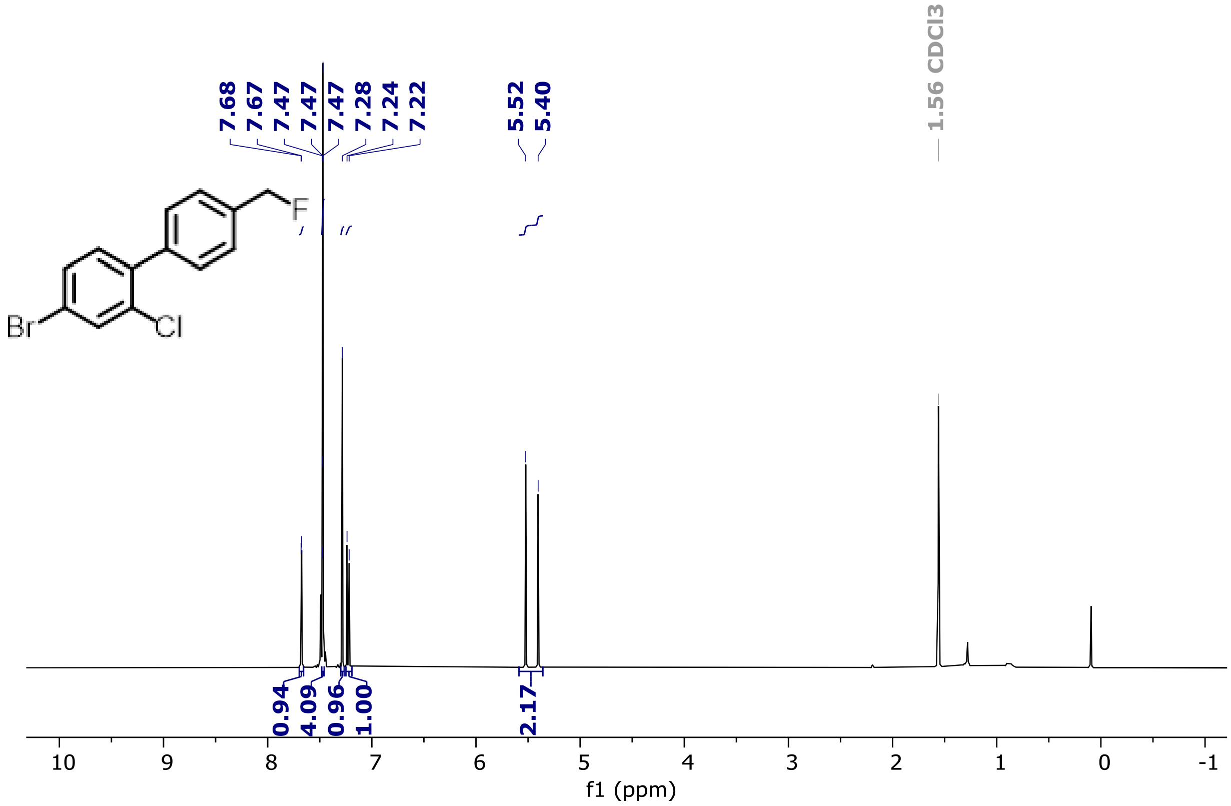

^{19}F NMR (377 MHz, $CDCl_3$)

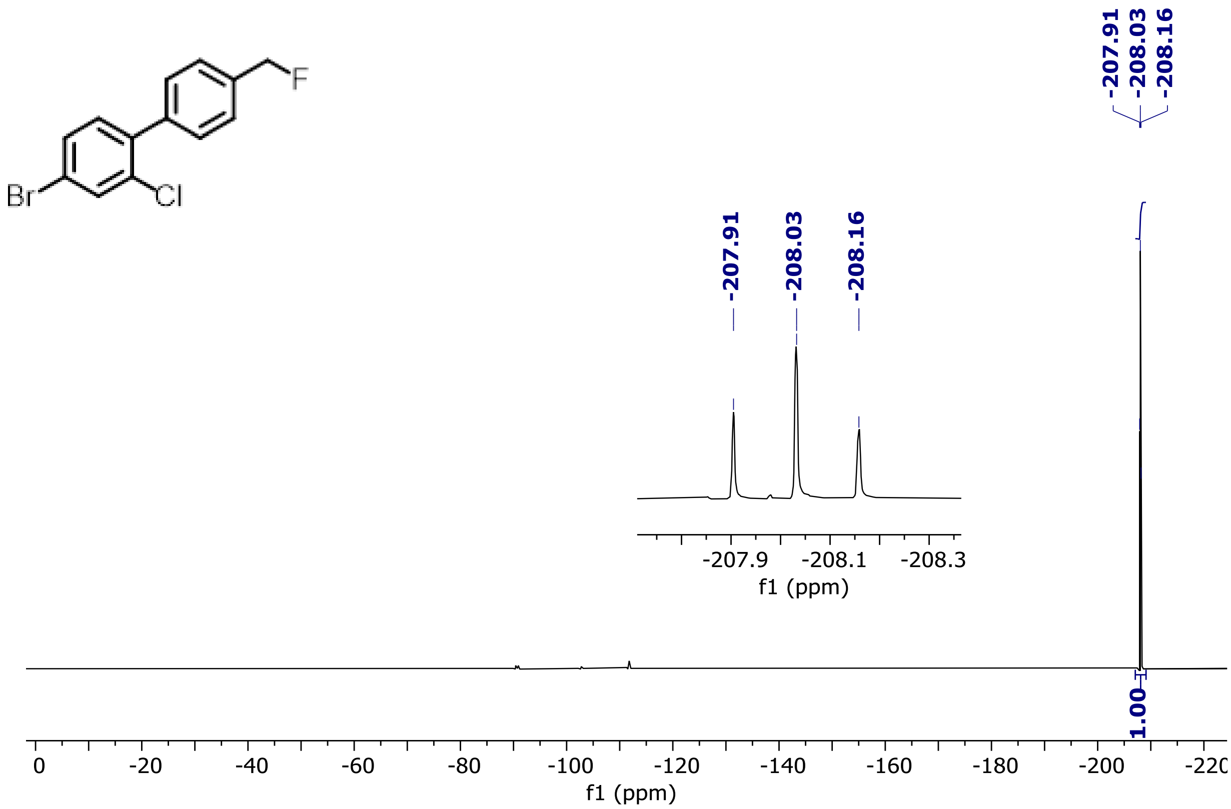

^{13}C NMR (101 MHz, $CDCl_3$)

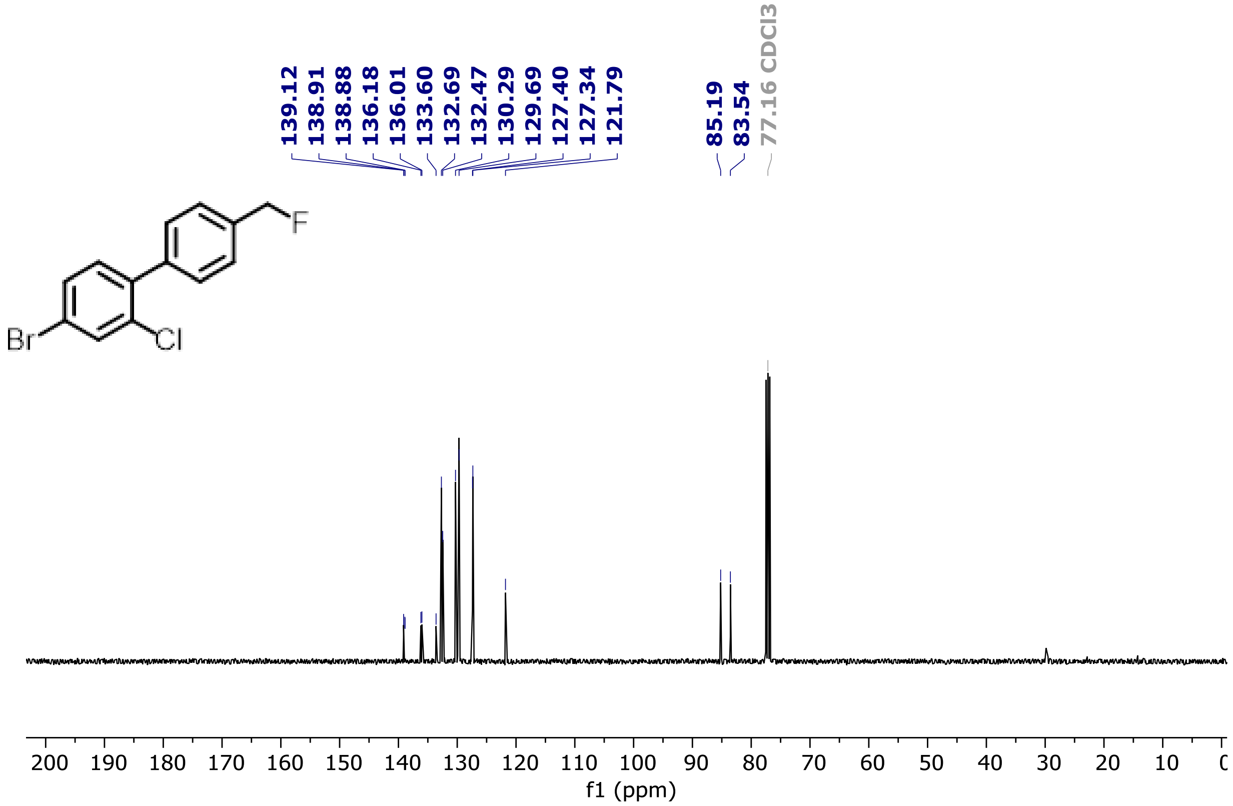

*1-Fluoro-2,3-dihydro-1H-indene **2i***

1-Fluoro-2,3-dihydro-1H-indene was prepared from 2,3,-dihydro-1H-indene via general procedure X. The final product could not be isolated via column chromatography on silica gel. ^{19}F NMR shifts for determining NMR yields were therefore assigned based on previous literature reports identifying the benzyl fluoride shift in crude reaction mixtures.[7] A representative crude NMR is provided in support.

[Crude] **^{19}F {^{1}H} NMR** (377 MHz)

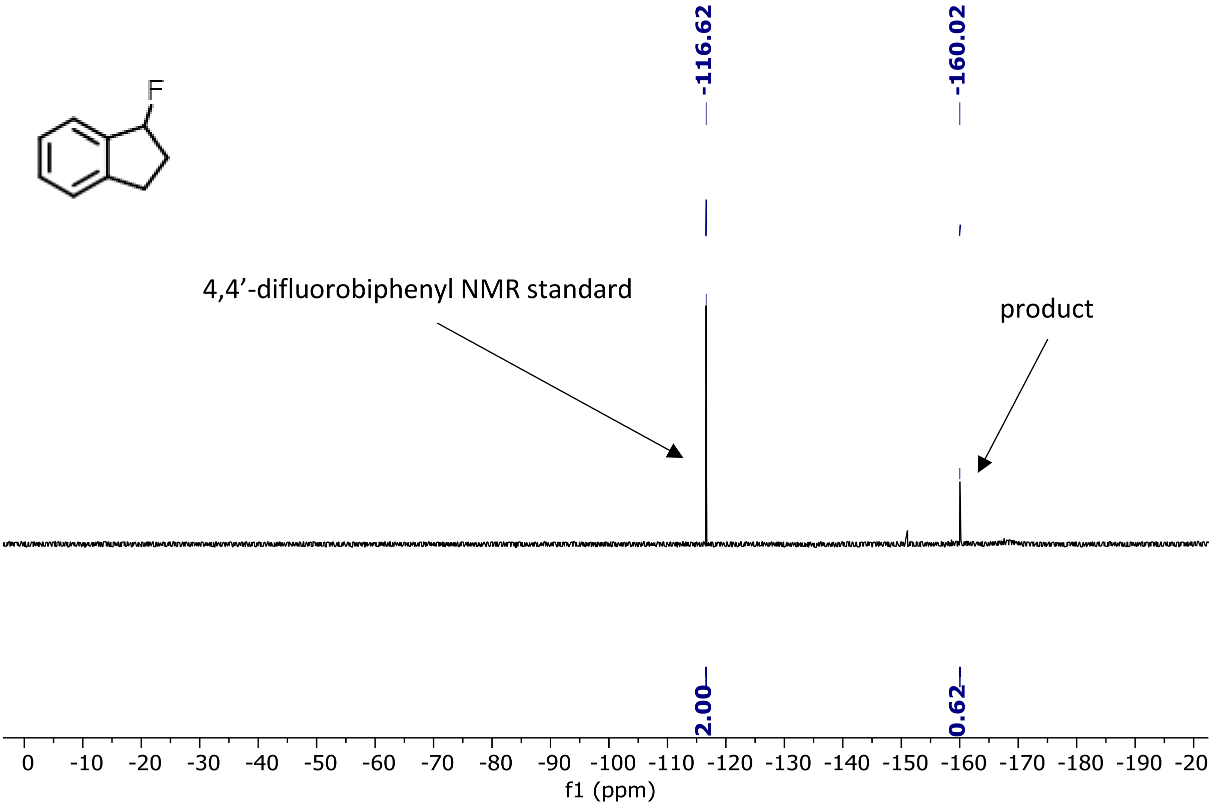

10. References

1 D. M. Heard and A. J. J. Lennox, *Angew. Chemie Int. Ed.*, 2020, **59**, 18866–18884.

2 M. Döbele, S. Vanderheiden, N. Jung and S. Bräse, *Angew. Chemie Int. Ed.*, 2010, **49**, 5986–5988.

3 S. I. Baker, M. Yaghoubi, S. L. Bidwell, S. L. Pierce, H. P. Hratchian and R. D. Baxter, *J. Org. Chem.*, 2022, **87**, 8492–8502.

4 Y. Zhou, W. You, K. B. Smith and M. K. Brown, *Angew. Chemie Int. Ed.*, 2014, **53**, 3475–3479.

5 Y. X. Luan, T. Zhang, W. W. Yao, K. Lu, L. Y. Kong, Y. T. Lin and M. Ye, *J. Am. Chem. Soc.*, 2017, **139**, 1786–1789.

6 J.-B. Xia, C. Zhu and C. Chen, *J. Am. Chem. Soc.*, 2013, **135**, 17494–17500.

7 A. Vasilopoulos, D. L. Golden, J. A. Buss and S. S. Stahl, *Org. Lett.*, 2020, **22**, 5753–5757.

8 S. Doobary, A. T. Sedikides, H. P. Caldora, D. L. Poole and A. J. J. Lennox, *Angew. Chemie Int. Ed.*, 2020, 59, 1155–1160.
